# *Lactobacillus reuteri* targets farnesoid X receptor-dependent bile acid homeostasis to ameliorate alcoholic liver disease

**DOI:** 10.1093/ismejo/wrag153

**Published:** 2026-06-16

**Authors:** Shuo Yuan, Yuhua Liu, Peng Gao, Jiaxu Sun, Yuanshi Wang, Zhiyong Du, Jun Lei, Jiaxin Hong, Xinlan Fang, Pengfei Tu, Yingyuan Lu, Yong Jiang

**Affiliations:** State Key Laboratory of Natural and Biomimetic Drugs, School of Pharmaceutical Sciences, Peking University, No. 38, Xueyuan Road, Haidian District, Beijing 100191, China; Beijing Institute of Heart Lung and Blood Vessel Disease, Beijing Anzhen Hospital, Capital Medical University, No. 2, Anzhen Road, Chaoyang District, Beijing 100029, China; State Key Laboratory of Natural and Biomimetic Drugs, School of Pharmaceutical Sciences, Peking University, No. 38, Xueyuan Road, Haidian District, Beijing 100191, China; State Key Laboratory of Natural and Biomimetic Drugs, School of Pharmaceutical Sciences, Peking University, No. 38, Xueyuan Road, Haidian District, Beijing 100191, China; Department of Gastroenterology, Affiliated Hospital of Beihua University, No. 12, Jiefang Middle Road, Chuanying District, Jilin City, Jilin Province 132011, China; Beijing Institute of Heart Lung and Blood Vessel Disease, Beijing Anzhen Hospital, Capital Medical University, No. 2, Anzhen Road, Chaoyang District, Beijing 100029, China; State Key Laboratory of Natural and Biomimetic Drugs, School of Pharmaceutical Sciences, Peking University, No. 38, Xueyuan Road, Haidian District, Beijing 100191, China; State Key Laboratory of Natural and Biomimetic Drugs, School of Pharmaceutical Sciences, Peking University, No. 38, Xueyuan Road, Haidian District, Beijing 100191, China; State Key Laboratory of Natural and Biomimetic Drugs, School of Pharmaceutical Sciences, Peking University, No. 38, Xueyuan Road, Haidian District, Beijing 100191, China; State Key Laboratory of Natural and Biomimetic Drugs, School of Pharmaceutical Sciences, Peking University, No. 38, Xueyuan Road, Haidian District, Beijing 100191, China; State Key Laboratory of Natural and Biomimetic Drugs, School of Pharmaceutical Sciences, Peking University, No. 38, Xueyuan Road, Haidian District, Beijing 100191, China; State Key Laboratory of Natural and Biomimetic Drugs, School of Pharmaceutical Sciences, Peking University, No. 38, Xueyuan Road, Haidian District, Beijing 100191, China

**Keywords:** *Lactobacillus reuteri*, farnesoid X receptor, bile acid metabolism, alcoholic liver disease, echinacoside

## Abstract

Alcoholic liver disease (ALD) is characterized by gut dysbiosis, yet the functional contribution of specific commensals remains largely unexplored. Here, we identify a significant reduction of *Lactobacillus reuteri* in both ALD patients and mouse models compared with their respective healthy controls. Using fecal microbiota transplantation, we first establish a causative role of the dysbiotic gut microbiota in driving ALD pathophysiology. In line with this finding, oral supplementation with *L. reuteri* ameliorates ALD phenotypes. Mechanistically, *L. reuteri* activates the farnesoid X receptor (FXR) signaling pathway, thereby restoring bile acid homeostasis, as evidenced by reduced levels of cholic acid (CA) and deoxycholic acid (DCA). The functional importance of FXR is further validated in *Fxr*^−/−^ mice, where the protective effects of *L. reuteri* on both ALD and bile acid metabolism are largely abrogated. Moreover, both *in vitro* and *in vivo* experiments confirm that CA and DCA directly compromise hepatocyte viability and exacerbate liver injury, reinforcing their roles as pathogenic effectors downstream of FXR dysregulation. Expanding upon these mechanistic insights, we identify the natural compound echinacoside as a gut microbiota-targeting intervention that not only enriches *L. reuteri* but also potentiates FXR signaling, leading to significant improvement of ALD. Collectively, our findings define a microbiota-host metabolic axis centered on *L. reuteri* and FXR-dependent bile acid homeostasis, offering a conceptual framework for targeting host–microbe interactions in ALD.

## Graphical Abstract

**Graphical Abstract:**
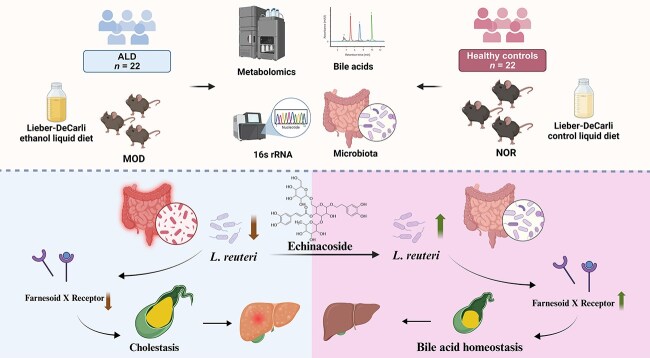

## Introduction

Alcoholic liver disease (ALD) is a progressive condition resulting from alcohol consumption, encompassing a spectrum of liver disorders that range from asymptomatic hepatic steatosis to hepatitis, liver fibrosis, and ultimately cirrhosis [1]. Despite its singular etiological cause, its pathogenesis is complex, and no targeted and reliable medical treatment is currently available [2, 3]. Mounting evidence has identified the “gut–liver axis” as a critical driver of ALD progression, propelling the exploration of gut microbiome-targeted preventive and therapeutic strategies to the forefront of liver disease research [4–6]. Central to this effort is the need to understand how gut microbial communities and their metabolic functions integrate with host signaling pathways—a fundamental question in microbial systems ecology.

The gut microbiome has emerged as a critical regulator of host health, and microbiome-modulating therapies have gained substantial attention for the management of various diseases. Probiotics, prebiotics, synbiotics, and fecal microbiota transplantation (FMT) have demonstrated efficacy in restoring microbial balance and improving clinical outcomes [7]. Recent advances highlight the therapeutic potential of microbiome-based interventions in ALD, particularly the protective effects of *Lactobacillus* and *Bifidobacterium* species [8–10]. Previous studies had shown that under the same conditions of alcohol-induced damage after ALD modeling, the symptoms of liver injury were milder in germ-free mice than in normal mice [11, 12]. These studies underscore the importance of microbiota–host crosstalk in ALD and provide a foundation for identifying microbiome-targeting agents.

Despite progress, clinical studies focusing on ALD patient samples remain limited, and there is a lack of in-depth mechanistic research on ALD pathogenesis directly based on clinical patients. Nevertheless, a few clinical and experimental animal studies have found that the abundance of several gut microbiota, such as *Bacteroidetes, Lactobacillus, Blautia*, and *Bifidobacterium* are significantly reduced in ALD patients and model animals [4, 13, 14]. Among these, *Lactobacillus reuteri* plays an important role in human health, with documented effects on liver disease, cancer, cardiovascular disease, and inflammatory bowel disease [15–19]. Previous studies have revealed that *L. reuteri* reversed the phenotype of ethanol-induced hepatitis and fatty acid metabolism disorders [20]; however, the detailed mechanisms behind this effect remain elusive.

A key player in bile acid homeostasis is the farnesoid X receptor (FXR), the first discovered bile acid receptor, which plays a crucial role in maintaining bile acid homeostasis and regulating metabolic processes [21, 22]. Activation of FXR in the liver can prevent triglyceride (TG) elevation and fatty degeneration, whereas the activation of FXR in the intestine can inhibit mucosal damage [23]. Conversely, inhibition of FXR has also been reported to significantly improve liver fibrosis [24]. Recent work has preliminarily demonstrated that *L. reuteri* and its postbiotics ameliorate hepatic lipid accumulation and fibrosis at least in part through modulation of FXR-related signaling pathway [25, 26]. However, it is unclear whether FXR is a key therapeutic target for *L. reuteri* in the treatment of ALD, and the relationship among *L. reuteri*, FXR, and bile acids is still poorly defined. FXR serves as the central sensor of bile acids, functioning to inhibit bile acid synthesis and promote bile acid excretion [27]. Bile acids, which are synthesized by the liver and metabolized through the intestine, are the most closely connected signaling molecules connecting the gut and the liver and play the most important role in bidirectional communication [28]. Although cholestasis is a classic pathological feature of ALD, current research on the hepatotoxicity of bile acids in this context remains insufficient.

Numerous studies have documented that traditional Chinese medicines and their active compounds can effectively modulate gut microbiota to exert therapeutic effects [29, 30]. Echinacoside (ECH), the main component in the famous traditional Chinese medicine Cistanches Herba (*Cistanche deserticola* and *Cistanche tubulosa*), *Echinacea angustifolia*, and *Ligustrum lucidum*, exhibits neuroprotective, reproductive protective, antioxidant, anti-inflammatory, and hepatoprotective effects [31–33]. Previous studies have demonstrated the therapeutic potential of ECH against alcohol-induced liver injury, although the underlying mechanisms remain incompletely understood [30, 34]. Given these findings, it is warranted to investigate whether ECH may function as a prebiotic to ameliorate ALD.

Consistent with the central question of microbial systems ecology, we herein systematically investigate the functional interplay between the gut microbiota, bile acid metabolism, and host FXR signaling in ALD by integrating clinical cohort analysis, multi-omics, and mechanistic *in vivo* validation. Specifically, we identify *L. reuteri* as a key commensal depleted in ALD and demonstrate that its restoration reshapes the gut microbial ecosystem and reprograms the bile acid pool to activate FXR signaling. Furthermore, we uncover ECH as a natural prebiotic that enriches *L. reuteri*, thereby revealing a microbe–host metabolic axis that can be therapeutically targeted. Collectively, our findings illustrate how a single commensal orchestrates ecosystem-level changes to influence host physiology, providing a framework for understanding microbiota–host interactions in disease.

## Materials and methods

### Patient enrollment and sample collection

This study recruited 22 healthy controls (HCs) and 22 ALD patients from the Affiliated Hospital of Beihua University, with all research procedures strictly adhering to medical ethics standards and approved by the Ethics Review Committee of Beihua University (Approval No.: 20240081). ALD patients met diagnostic criteria including chronic alcohol use with abnormal liver function and mild to moderate alcoholic hepatitis, whereas they were excluded for concurrent liver diseases such as hepatitis B or fatty liver disease and recent acute liver failure or antibiotic use. HCs were required to be age- and sex-matched with the ALD group, absence of liver disease or gastrointestinal disorders, and have no recent antibiotic/probiotic use and alcohol consumption within 3 months, with exclusions for comorbid metabolic diseases like diabetes or obesity and recent immunosuppressant use.

Clinical sample collection was performed as follows: blood samples (5 ml per subject) were collected in serum separator tubes, allowed to clot for 30 min at room temperature, and centrifuged at 3000 × g for 10 min to obtain serum, which was aliquoted and stored at −80°C; fresh fecal specimens (≥200 mg per sample) were collected in sterile tubes, immediately frozen on dry ice, and transferred to −80°C within 2 h for long-term storage until analysis.

### Animals and experimental design

Male C57BL/6 mice (6-week-old, body weight 20 ± 2 g) were obtained from the Department of Laboratory Animal Science, Peking University Health Science Center [production license No. SCXK (Jing) 2021-0013], with all experimental procedures conducted in strict compliance with the Institutional Animal Care and Use Committee guidelines of Peking University (approval No. LA2021231). The *Fxr*^−/−^ mice were originally generated by Mouse-Saving Biosciences, Beijing. The animals were divided into cages randomly, with four mice per cage under specific pathogen-free (SPF) conditions, and provided with standard diet and water *ad libitum* throughout the acclimatization and experimental periods.

Animal modeling and drug administration: Following 1 week of acclimatization under standard conditions, 24 mice were randomly divided into groups (*n* = 12). The ALD model was constructed according to the protocol of the National Institute on Alcohol Abuse and Alcoholism (NIAAA) model [35]. The detailed method is listed in Supplementary Information S1.1.

Fecal microbiota transplantation: Twenty-four mice were initially acclimated in SPF for 7 days, followed by a 5-day daily oral gavage regimen of an antibiotic cocktail (vancomycin 100 mg/kg, neomycin sulfate 200 mg/kg, metronidazole 200 mg/kg, and ampicillin 200 mg/kg) to establish pseudo-germ-free animals. On the fifth day, the pseudo-germ-free mice were randomly divided into two groups (*n* = 12): one group of the mice was treated with normal group feces (FMT-NOR) and the other group was treated with model group feces (FMT-MOD). Then the mice received daily oral gavage of 200 μl FMT suspension prepared from either NOR or MOD donor mice for 4 consecutive weeks, with the FMT solution prepared by homogenizing fresh fecal pellets in sterile phosphate-buffered saline (PBS) at a concentration of 500 mg/ml, followed by sequential filtration through 100 and 40 μm cell strainers to remove particulate matter and centrifugation at 1000 × g for 10 min to obtain a clear bacterial suspension.

The effect of *L. reuteri* monocolonization in ALD mice: Following weighing, 48 mice were randomly divided into four groups (*n* = 12): NOR, MOD, *L. reuteri* monocolonization (Lr) group, and heat-killed *L. reuteri* (HKL) group. The bacterial culture was centrifuged at 4°C and 4000 × g for 30 min after which the supernatant was discarded. The pellet was washed twice with sterile PBS and re-suspended in sterile PBS to prepare a bacterial suspension with a final concentration of 1 × 10^9^  colony-forming unit (CFU)/ml for monocolonization. HKL was heated at 105°C for 30 min. The MOD, Lr, and HKL groups were subjected to the chronic ALD model establishment. The Lr and HKL groups received a daily oral gavage of 200 μl Lr or HKL suspension, whereas the NOR and MOD groups received 200 μl of physiological saline as controls.

The effect of bile acids in ALD mice: Forty-eight male C57BL/6 mice were randomly divided into four groups (*n* = 12): NOR, MOD, model mice treated with cholic acid (CA) (100 mg/kg) group, and model mice treated with deoxycholic acid (DCA) (100 mg/kg) group.

The effect of bile acids in normal mice: Thirty-six male C57BL/6 mice were randomly divided into three groups (*n* = 12): NOR, normal mice treated with CA (NOR + CA), and normal mice treated with DCA (NOR + DCA). The bile acids were uniformly mixed into the standard chow at a concentration of 0.1% (*w/w*), and all experimental groups receiving equal amounts of feed daily during the 25-day feeding period. The diet was prepared fresh weekly under sterile conditions to ensure compound stability, and feed intake was monitored daily to confirm consistent consumption across groups (mean intake: 2.5 ± 0.3 g/day/mouse). The other treatments were the same as before.

The effect of bile acids in normal *Fxr^−/−^* mice: Thirty *Fxr^−/−^* mice were randomly divided into three groups (*n* = 10). The other treatments were the same as the normal mice.

The effect of Lr in wild type or *Fxr^−/−^* ALD mice: Wild type mice and *Fxr^−/−^* knockout mice were respectively and randomly divided into three groups (*n* = 10): NOR, MOD, and Lr group.

The effect of ECH in ALD mice: Sixty male C57BL/6 mice were randomly divided into six groups (*n* = 10): NOR, MOD, low-dose ECH (ECH 50, 50 mg/kg), medium-dose ECH (ECH 100, 100 mg/kg), high-dose ECH (ECH 200, 200 mg/kg), and positive drug bifendate (100 mg/kg).

### Sample collection

Following the experimental period, blood samples were obtained from anesthetized mice through orbital venous plexus puncture with heparinized capillary tubes. The animals were then euthanized by cervical dislocation under deep anesthesia to ensure death. Subsequently, the liver and small intestine were promptly excised. Liver tissues were perfused with ice-cold normal saline to remove residual blood. Fresh fecal pellets were collected from the cecum into sterile tubes. All collected samples were immediately snap-frozen in liquid nitrogen. The plasma samples were obtained from blood samples by centrifugation (4000 rpm, 10 min, 4°C).

### Histopathological evaluation

For each experimental group, three mice were randomly selected for liver and small intestine tissue collection. The right hepatic lobe and caudate lobe were surgically excised, promptly washed thoroughly with PBS that had been pre-chilled on ice, and immediately immersion-fixed in freshly prepared, precooled (4°C) 4% paraformaldehyde solution for optimal tissue preservation. Following fixation, the liver caudate lobe specimens were cryosectioned at 10 μm thickness using a precision freezing microtome (Model JT-12S, Wuhan Junjie Electronics Co., Ltd, China) maintained at −20°C. Tissue sections underwent three sequential 5-min PBS washes to remove residual fixative before being incubated with filtered Oil Red O working solution (Sigma-Aldrich, O0625) for precisely 12 min at room temperature (22 ± 1°C) under light-protected conditions. Sections were then differentiated in 60% isopropanol (*v/v*) with microscopic monitoring until complete stromal clearance was achieved, followed by rigorous rinsing with distilled water. Nuclear counterstaining was performed using Mayer’s hematoxylin (5 min) with subsequent bluing in running tap water, and slides were permanently mounted using a glycerol-gelatin mounting medium. Concurrently, the remaining liver tissues underwent graded ethanol dehydration (70%, 85%, 95%, and 100%), xylene clearing, and paraffin infiltration in a temperature-controlled wax-dissolving chamber (58 ± 1°C) for 4 h prior to embedding. The resulting paraffin blocks were sectioned at a 5 μm thickness using a calibrated rotary microtome (RM2235, Leica Microsystems) for subsequent hematoxylin (5 min) and eosin (1 min) staining following standard protocols. All histological procedures included appropriate positive and negative controls to ensure staining specificity and reproducibility. Immunofluorescence analysis is provided in Supplementary Information S1.2.

### Determination of biochemical parameters

Randomly selected liver samples from each group were homogenized (1:5, *w/v*) in normal saline and centrifuged to obtain the supernatant. Biochemical parameters in the supernatant, including alanine aminotransferase (ALT), aspartate aminotransferase (AST), TG, tumor necrosis factor-α (TNF-α), interleukin-1β (IL-1β), glutathione peroxidase (GSH-Px), malondialdehyde (MDA), and superoxide dismutase (SOD) were quantified using specific commercial assay kits according to the instructions. All assays included standard curves and were performed in duplicate.

### 16S ribosomal ribonucleic acid (rRNA) gene sequencing

Microbial genomic DNA was extracted from fecal samples, and the 16S rRNA gene was sequenced by Biotree Co. Ltd (Shanghai, China). Detailed experimental procedures are provided in Supplementary Information S1.3.

### Targeted metabolomics (T700)

The targeted metabolomics assay was performed using the Maiwei T700 method developed by Wuhan Maiwei Metabolism Biotechnology Co., Ltd. The detail of method is listed in Supplementary information S1.4.

### Bile acid-targeted metabolomics of human serum

For the serum of human, aliquots of specimens were transferred to 2-ml centrifuge tubes, followed by the precise addition of 400 μl of pre-chilled methanol (−20°C). The detail of method is listed in Supplementary information S1.5.

### Bile acid-targeted metabolomics of mouse liver

Liver samples were thawed gradually from −20°C to 0°C. Precisely 50 mg of tissue was weighed and homogenized. The detail of method is listed in Supplementary information S1.6.

### Cell culture and viability

HepG2 and L02 cell lines were bought from the Cell Bank of Peking Union Medical College (Beijing, China), and were maintained in high-glucose Dulbecco's Modified Eagle Medium (DMEM) supplemented with 10% fetal bovine serum at 37°C with 5% CO_2_. For assays, cells were seeded in 96-well plates (4000 cells/well) adhered overnight, and treated with drugs for 24 h. Lr and HKL supernatants, prepared by anaerobic culture and 0.22-μm filtration, were among the treatments. Cell viability was then assessed by 3-(4,5-dimethylthiazol-2-yl)-2,5-diphenyltetrazolium bromide (MTT) assay.

### Real-time fluorescence quantitative polymerase chain reaction (PCR)

Following isolation with a commercial kit and NanoDrop quantification, complementary DNA (cDNA) was generated from total RNA employing a commercial RT Master Mix. Gene expression analysis was then conducted via SYBR Green-based quantitative real-time polymerase chain reaction (qRT-PCR), normalizing to glyceraldehyde-3-phosphate dehydrogenase (GAPDH) [36]. All primer sequences utilized in this study can be found in Supplementary Table S5.

### Western blotting

Protein extraction was performed using NP-40 buffer supplemented with 1% protease inhibitors, and concentrations were determined via a bicinchoninic acid (BCA) assay prior to separation by sodium dodecyl sulfate-polyacrylamide gel electrophoresis (SDS-PAGE). The separated proteins were then transferred to polyvinylidene difluoride (PVDF) membranes. The membranes were immunoblotted overnight at 4°C with specific primary antibodies: anti-FXR (67778-1-Ig, Proteintech) and anti-GAPDH (60004-1-Ig, Proteintech). This was followed by incubation with horseradish peroxidase (HRP)-conjugated secondary antibodies and detection using an enhanced chemiluminescence (ECL) system.

### Bacterial growth curve assay

*L. reuteri* was cultured anaerobically in Gifu anaerobic medium (GAM) broth, and a modified GAM broth without glucose and soluble starch (Qingdao Hope Bio-Technology Co., Ltd, China). The details of the method are listed in Supplementary Information S1.7.

### Statistical analysis

The liver index (liver weight/body weight × 100%) and other continuous data are expressed as mean ± SD. Statistical analyses were performed using GraphPad Prism (version 9.0). Between-group comparisons were assessed using an unpaired two-tailed Student’s *t-*test (or one-way assessed using one-way analysis of variance (ANOVA), as appropriate). A two-tailed *P*-value of <.05 was considered statistically significant. Correlation analysis, network construction, and analysis of covariance (ANCOVA) are listed in Supplementary information S1.8 and S1.9.

## Results

### Gut microbiota imbalance occurs in both alcoholic liver disease patients and mice

**Figure 1:**
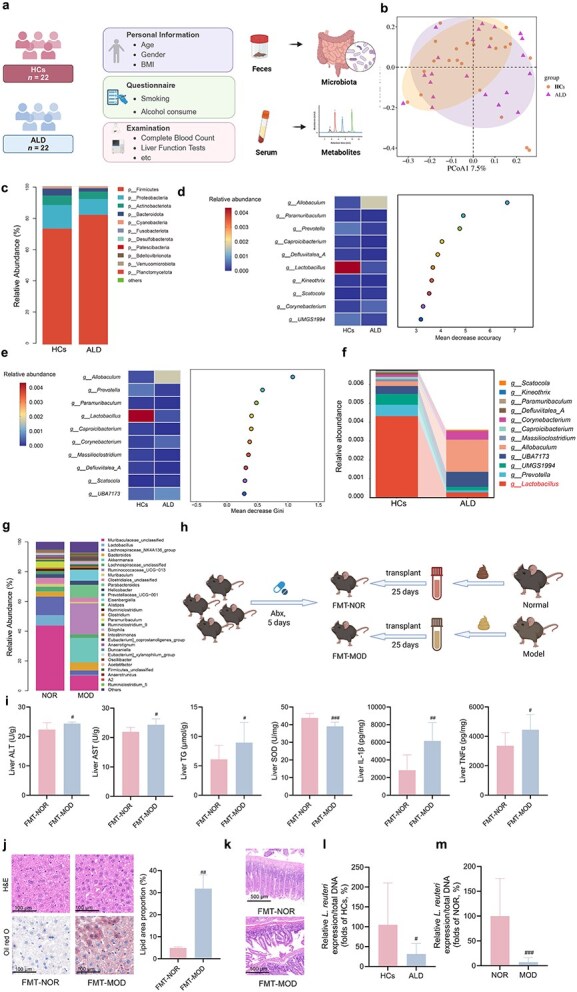
Gut microbiota imbalance occurs in both ALD patients and mice (a) experimental procedure of HCs and ALD; (b) PCoA plot of fecal samples between HCs and ALD; (c) relative abundance of gut microbiota at the phylum level between HCs and ALD; (d) relative abundance and mean decrease accuracy of top 10 genus markers between HCs and ALD identified by random forest classifier; (e) relative abundance and mean decrease Gini of 10 genus markers between HCs and ALD identified by random forest classifier; (f) relative abundance of potential markers at the genus level between HCs and ALD identified by random forest classifier; (g) relative abundance of gut microbiota at the genus level between NOR and MOD; (h) experimental procedure of FMT; (i) liver ALT, AST, TG, SOD, IL-1β, and TNF-α levels in each group (*n* = 8); (j) H&E staining and oil red O staining of live tissues in each group (*n* = 3), scale bar =100 μm; (k) H&E staining of small intestine tissues in each group (*n* = 3), scale bar =50 μm; (l) the relative abundance of *L. reuteri* in feces of HCs and ALD by qRT-PCR; (m) the relative abundance of *L. reuteri* in feces of NOR and MOD by qRT-PCR; HCs: healthy controls; ALD: ALD patients; NOR: normal group; MOD: model group; FMT-NOR: normal mice treated with NOR feces; FMT-MOD: normal mice treated with MOD feces; compared with NOR or HCs or FMT-NOR; ^#^*P <* .05, ^##^*P <* .01, ^###^*P <* .001.

Clinical baseline data for the patients and HCs are provided in Supplementary Table S6. Fecal samples from both HCs and ALD patients were subjected to 16S rRNA gene sequencing, whereas serum samples were subjected to metabolomics (Fig. 1a). A principal co-ordinates analysis (PCoA) plot was performed to assess beta diversity (Fig. 1b), reflecting compositional differences in the gut microbiota between the two groups. The results revealed a clear separation between HCs and ALD patients, suggesting significant alterations in microbial community composition. At the phylum level, the gut microbiota of HCs was primarily composed of Firmicutes, Bacteroidota, Actinobacteriota, and Proteobacteria, whereas ALD patients showed an increase in Firmicutes and a decrease in Bacteroidota, Actinobacteriota, and Proteobacteria (Fig. 1c). The ratio of Firmicutes/Bacteroidota was increased nearly three-fold, which was consistent with a previous study [37]. At the genus level, a random forest classifier was employed to identify the most influential taxa. Among the top 10 genera ranked by mean decrease accuracy and mean decrease Gini, *Allobaculum, Prevotella, Paramuribaculum*, and *Lactobacillus* exhibited consistently high importance scores and were significantly more abundant in HCs (Fig. 1d and e). Bar plot analysis further revealed that *Lactobacillus* had the highest relative abundance among these genera in HCs (Fig. 1f). Collectively, these results identify *Lactobacillus* as a key discriminant taxon associated with healthy status and a promising candidate for probiotic intervention.

Significant differences in alpha and beta diversity were observed between ALD and control mice (Supplementary Figure S1A and B). The NOR mice were dominated by Bacteroidetes, whereas the MOD mice exhibited increased proportions of Firmicutes, Verrucomicrobia, Proteobacteria, and Epsilonbacteraeota, alongside a reduction in Bacteroidetes (Supplementary Fig. S1C). Stacked bar plots at the genus level were used to identify differential microbial taxa. Long-term ethanol intake led to a decrease in the abundance of *Lactobacillus, Lachnospiraceae_NK4A136_group, Muribaculum, Alistipes*, and *Paramuribaculum*, whereas *Akkermansia, Ruminococcaceae_UCG-013, Parabacteroides, Eisenbergiella, Bilophila*, and *Ruminiclostridium_9* were increased (Fig. 1g).

Hematoxylin and eosin (H&E) staining of small intestinal tissues from NOR and MOD mice showed that the intestinal villi of the NOR group were well-organized and tightly connected to the intestinal wall, whereas in the MOD group, ethanol-induced damage resulted in thinning of the intestinal wall, morphological damage to the villi, and the appearance of gaps between the villi and the intestinal wall (Supplementary Figure S1D). Lipopolysaccharide (LPS) levels were significantly elevated in the MOD group (*P <* .05), indicating that ethanol-induced damage caused intestinal barrier leakage, thereby allowing LPS to enter the bloodstream (Supplementary Figure S1E). Immunohistochemical staining for Occludin in the small intestine (Supplementary Figure S1F) revealed high expression in the NOR group, whereas Occludin expression was reduced in the MOD group.

To further confirm the involvement of gut microbiota in the pathophysiology of ALD, an FMT experiment was conducted. Fecal microbiota from the NOR and MOD groups was collected and transplanted via oral gavage into recipient mice whose gut microbiota had been depleted by broad-spectrum antibiotics, resulting in the FMT-NOR and FMT-MOD groups (Fig. 1h). The results showed that FMT-MOD mice exhibited significant liver injury, as evidenced by higher ALT, AST, TG, and inflammatory factors such as TNF-α and IL-1β, and lower SOD compared to the FMT-NOR group (Fig. 1i). Liver histopathological scores were also higher in the FMT-MOD group, whereas the barriers of small intestinal were damaged (Fig. 1j and k). The results of FMT demonstrate that the pathogenesis of ALD is closely associated with gut microbiota, thereby providing a rationale for the potential of probiotic-based therapies in ALD treatment.

We observed a significant reduction in the genus *Lactobacillus* in both ALD patients and experimental models, consistent with established patterns of microbial dysbiosis in alcohol-related liver injury. Prior studies identify *L. reuteri* as a functionally distinct species within this genus, demonstrating pronounced hepatoprotective and lipid-regulating capabilities in ALD pathogenesis [20, 25]; we thus prioritized *L. reuteri* for targeted analysis to elucidate its relationship with the clinical and murine ALD phenotype and mechanisms. The abundance of *L. reuteri* was detected using qRT-PCR with specific primers (Fig. 1l and m), and the results confirmed a significant reduction of *L. reuteri* in both ALD patients and model mice. Overall, these findings demonstrated that ALD significantly altered gut microbiota composition, particularly by reducing the abundance of *L. reuteri.*

### Effects of *Lactobacillus reuteri* single bacterial colonization in mice

**Figure 2:**
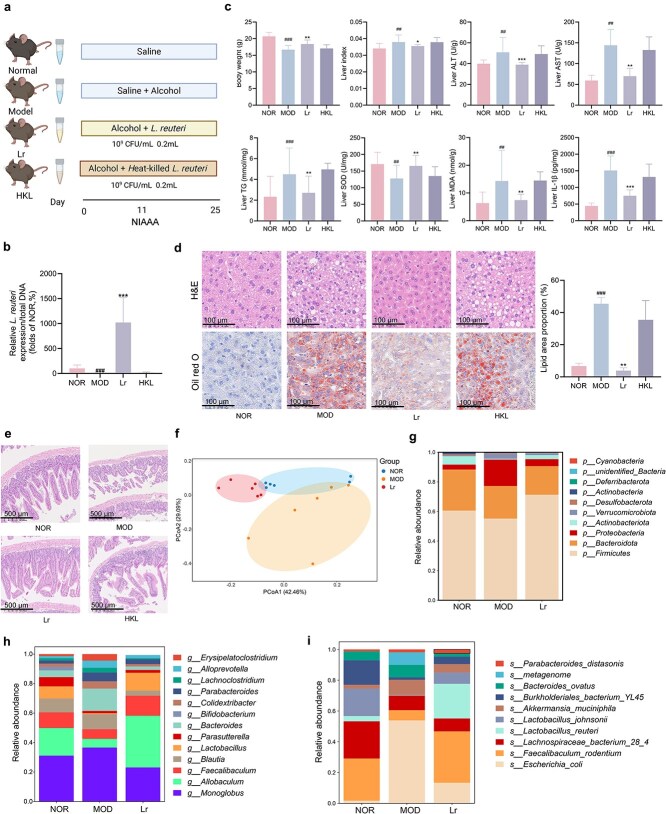
Effects of *L. reuteri* colonization in ALD mice; (a) experimental demonstration; (b) relative abundance of *L. reuteri* in fecal samples; (c) body weight, liver index, ALT, AST, TG, SOD, MDA, and IL-1β levels in each group (*n* = 8); (d) H&E staining and oil red O staining of liver in each group (*n* = 3), scale bar = 100 μm; (e) H&E staining of small intestine tissues in each group (*n* = 3), scale bar = 500 μm; (f) PCoA plot of fecal samples in each group (*n* = 6); (g) relative abundance of main gut microbiota at the phylum level in each group (*n* = 6); (h) relative abundance of main gut microbiota at the genus level in each group (*n* = 6); (i) relative abundance of main gut microbiota at the species level in each group (*n* = 6); NOR: normal group; MOD: model group; Lr: ALD mice treated with *L. reuteri*; HKL: ALD mice treated with heat-killed *L. reuteri*; compared with NOR, ^#^*P <* .05, ^##^*P <* .01, ^###^*P <* .001; compared with MOD, ^*^P < .05, ^**^P < .01, and ^***^P < .001.

To investigate the role of *L. reuteri* in ALD, a supplementation experiment was performed (Fig. 2a). The qPCR analysis using species-specific primers confirmed the successful colonization of *L. reuteri* and revealed a significant increase in fecal *L. reuteri* abundance in the *L. reuteri*-treated group compared with the MOD group (Fig. 2b). The results demonstrated that *L. reuteri* supplementation significantly reduced liver index, serum ALT and AST levels, as well as decreased oxidative stress, and inflammatory markers in ALD mice. However, HKL had no significant protective effect (Fig. 2c). *L. reuteri* treatment significantly reduced liver histopathological scores compared to the MOD group (Fig. 2d). In addition, H&E staining of small intestinal sections revealed that alcohol-induced disruption of intestinal wall structure and villus integrity was significantly ameliorated by *L. reuteri* treatment (Fig. 2e). Furthermore, immunofluorescence staining showed that *L. reuteri* treatment restored the alcohol-induced reduction of tight junction proteins Occludin, ZO-1, and Claudin-1 (Supplementary Figure S2A–C). Overall, these findings suggest that *L. reuteri* can ameliorate ALD by protecting intestinal barrier integrity.

To further evaluate the impact of *L. reuteri* colonization on gut microbiota composition, 16S rRNA gene sequencing was performed on cecal contents from the NOR, MOD, and *L. reuteri* groups. A PCoA plot revealed a clear separation between the NOR and MOD groups, whereas the *L. reuteri* group clustered closely with the NOR group, indicating that *L. reuteri* intervention partially restored the alcohol-induced gut microbial dysbiosis (Fig. 2f). At the phylum level, *L. reuteri* supplementation significantly increased the relative abundance of Firmicutes (Fig. 2g). At the genus level, in addition to a marked increase in *Lactobacillus, L. reuteri* treatment also elevated the levels of other potentially beneficial genera, including *Allobaculum, Faecalibaculum*, and *Parasutterella* (Fig. 2h). Consistently, species-level analysis confirmed that *L. reuteri* was significantly enriched after *L. reuteri* supplementation, demonstrating successful engraftment and underscoring the modulatory role of *L. reuteri* in reshaping gut microbial composition. Moreover, other beneficial species, such as *Lactobacillus johnsonii* and *Faecalibaculum rodentium*, also showed increased abundance (Fig. 2i). Collectively, these results demonstrate that *L. reuteri* supplementation exerts a holistic regulatory effect on gut microbial composition, effectively counteracting alcohol-induced dysbiosis and promoting a beneficial microbiota profile.

To investigate the metabolic impact of *L. reuteri* supplementation, targeted metabolomic profiling of over 700 key endogenous metabolites—including short-chain fatty acids (SCFAs), amino acid derivatives, and bile acids—was performed on fecal samples. Partial least squares discriminant analysis (PLS-DA) revealed that *L. reuteri* treatment significantly improved the metabolic profile of fecal contents in the MOD group, shifting it toward that of the NOR, and permutation tests confirmed the absence of overfitting (Supplementary Figure S3A and B). Differential metabolite analysis identified bile acids, nucleotides and their derivatives, and amino acids and their derivatives as key discriminators between the NOR and MOD groups (Supplementary Figure S3C). The *L. reuteri* supplement reshaped the host metabolic landscape, with pronounced alterations in multiple classes of metabolites, including amino acids and their derivatives, bile acids, SCFAs, and organic acids (Supplementary Figure S3D). In parallel, immunofluorescence analysis showed that *L. reuteri* supplement slightly upregulated intestinal AhR expression, although no significant differences in GPR43 or AhR were observed between the NOR and MOD groups (Supplementary Figure S4).

### *Lactobacillus reuteri* activated farnesoid X receptor to alleviate alcoholic liver disease

**Figure 3:**
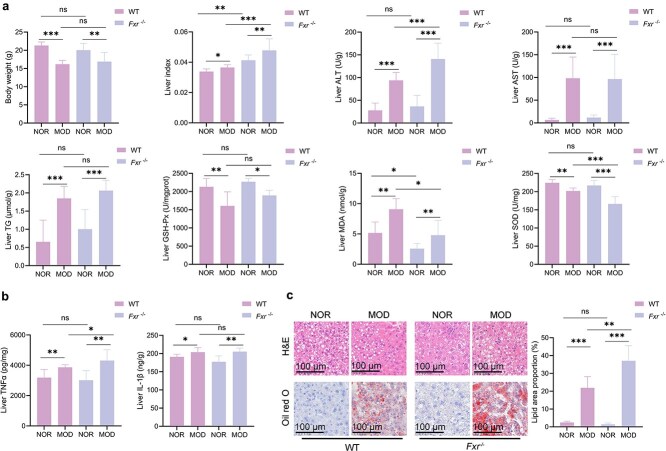
*Fxr* knockout exacerbates ALD; (a) body weight, liver index, liver ALT, AST, TG, GSH-Px, MDA, and SOD levels in WT and *Fxr^−/−^* mice (*n* = 10); (b) liver IL-1β and TNF-α levels in WT and *Fxr^−/−^* mice (*n* = 8); (c) H&E staining and oil red O staining of liver in WT and *Fxr^−/−^* mice (*n* = 3), scale bar = 100 μm; NOR: normal group; MOD: model group; ^ns^*P* ≥ .05, ^*^P < .05, ^**^P < .01, and ^***^P < .001.

FXR, a key member of the nuclear receptor superfamily, is predominantly expressed in the liver, intestine, and kidney. It serves as a central regulator of bile acid homeostasis and lipid metabolism [21]. The role of FXR in liver disease remains somewhat controversial. Some studies report that its activation can prevent TG elevation and fatty degeneration, whereas others suggest that inhibiting FXR may significantly improve liver fibrosis [23, 24]. To evaluate the relationship between FXR and ALD, we observed the liver injury in wild-type (WT) and *Fxr*^−/−^ mice after ALD modeling. The results showed that compared with WT mice, *Fxr*^−/−^ mice exhibited significantly greater liver damage, a higher liver index, higher ALT and AST levels, higher inflammatory factor levels, and higher histological scores after ALD modeling (Fig. 3a–c). These research findings demonstrate that FXR exerts hepatoprotective effects and can mitigate the development and progression of ALD.

**Figure 4:**
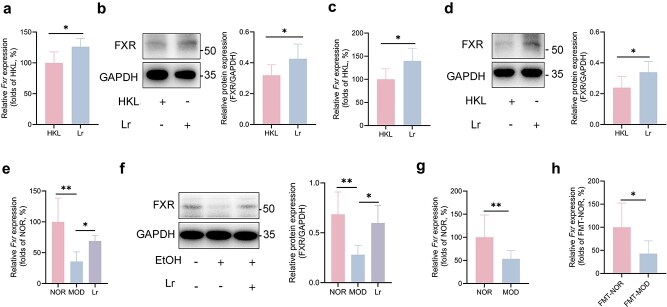
*L. reuteri* activates FXR; (a) the relative mRNA expression levels of *Fxr* in HepG2 cell (*n* = 6); (b) the protein expression levels of FXR in HepG2 cell (*n* = 4); (c) the relative mRNA expression levels of *Fxr* in L02 cell (*n* = 6); (d) the protein expression levels of FXR in L02 cell (*n* = 4); (e) the relative mRNA expression levels of *Fxr* in the mice liver (*n* = 6); (f) the protein expression levels of FXR in mice liver (*n* = 4); (g) the relative mRNA expression levels of *Fxr* in NOR and MOD mice liver (*n* = 6); (h) the relative mRNA expression levels of *Fxr* in FMT-NOR and FMT-NOR mice liver (*n* = 6); HKL: liver cell treated with the supernatant of heat-killed *L. reuteri*; Lr: liver cell or ALD mice treated with the supernatant of *L. reuteri*; NOR: normal group; MOD: model group; Lr: FMT-NOR: normal mice treated with NOR feces; FMT-MOD: normal mice treated with MOD feces; ^ns^*P* ≥ .05, ^*^P < .05, and ^**^P < .01.

A previous study suggested that the postbiotics of *L. reuteri* might alleviate ALD through modulating the FXR signaling pathway [26]. To further investigate whether *L. reuteri* regulates FXR to exert therapeutic effects on ALD, we added both *L. reuteri* supernatants and the inactivated *L. reuteri* to cell culture and observed that the *L. reuteri* supernatant could enhance both the transcriptional and protein expression levels of FXR in HepG2 and L02 cells (Fig. 4a–d and Supplementary Figure S5A and B), providing further evidence that *L. reuteri*-derived metabolites target and regulate FXR. Consistently, both the messenger ribonucleic acid (mRNA) and protein expression levels of FXR in the liver tissue of mice from the *L. reuteri* colonization group were significantly increased (Fig. 4e and f and Supplementary Figure S5C). Conversely, FXR mRNA expression levels were significantly decreased in the ALD mice group and in recipient mice receiving fecal transplantation from ALD mice (Fig. 4g and h). Taken together, these *in vitro* and *in vivo* results demonstrated that *L. reuteri* activates FXR.

To identify potential FXR agonists in *L. reuteri* culture supernatant, targeted metabolomics analysis was performed to compare the live *L. reuteri* culture supernatant with HKL culture supernatant as a control. Principal component analysis (PCA) plot and heatmap analyses revealed a clear separation between the two groups (Supplementary Figure S6A and B). Volcano plot analysis identified a total of 76 metabolites significantly enriched in the live bacterial supernatant, spanning multiple classes including amino acids and their metabolites, SCFAs, organic acid metabolites, and indole derivatives (Supplementary Figure S6C–E and Supplementary Table S7). Among these, indole derivatives, bile acids, and SCFAs have been previously reported to directly or indirectly activate FXR signaling [38–41]. Therefore, to further assess their potential as FXR agonists, metabolites with a fold change ˃10 were selected for further molecular docking using the FXR crystal structure (PDB:3BEJ). As a result, several of these metabolites were found to stably bind to the FXR ligand-binding pocket with Glide GScore values below −7.0 kcal/mol (Supplementary Table S8). Collectively, these results suggest that *L. reuteri*-derived metabolites may activate FXR, thereby regulating hepatic function.

**Figure 5:**
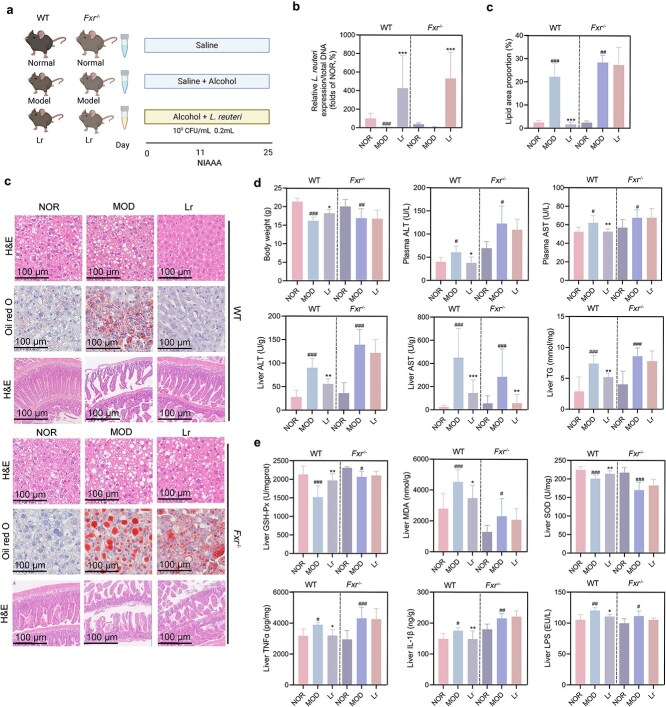
FXR serves as the critical mediator for *L. reuteri*-induced amelioration of ALD; (a) experimental demonstration; (b) relative abundance of *L. reuteri* in fecal samples (*n* = 10); (c) H&E staining of liver and small intestine tissues, and oil red O staining of liver in each group (*n* = 3), scale bar =100 μm; (d) body weight, ALT, AST, and TG levels in each group (*n* = 10); (e) GSH-Px, MDA, SOD, TNF-α, IL-1β, and LPS in each group (*n* = 8); NOR: normal group; MOD: model group; Lr: ALD mice treated with *L. reuteri*; compared with the NOR, ^#^*P <* .05, ^##^*P <* .01, ^###^*P <* .001; compared with the MOD, ^*^P < .05, ^**^P < .01, and ^***^P < .001.

To further verify the role of FXR in mediating the protective effects of *L. reuteri* against ALD, *L. reuteri* was orally administered to WT and *Fxr*^−/−^ ALD mice (Fig. 5a). The qPCR analysis revealed a significant increase in fecal *L. reuteri* abundance in the *L. reuteri*-treated group compared to the MOD group in both WT and *Fxr*^−/−^ ALD mice (Fig. 5b). In WT mice, *L. reuteri* supplementation significantly ameliorated alcohol-induced hepatic steatosis as assessed by oil red O staining, reduced liver histopathological scores, and alleviated intestinal damage (Fig. 5c). In contrast, these protective effects were absent in *Fxr*^−/−^ ALD mice. Moreover, *L. reuteri* failed to reduce liver index, ALT, AST and TG levels, oxidative stress, inflammatory factors, or LPS levels (Fig. 5d and e). Overall, these findings suggest that *L. reuteri* improves liver damage caused by ALD by targeting FXR.

### Bile acid-targeted metabolomics studies

**Figure 6:**
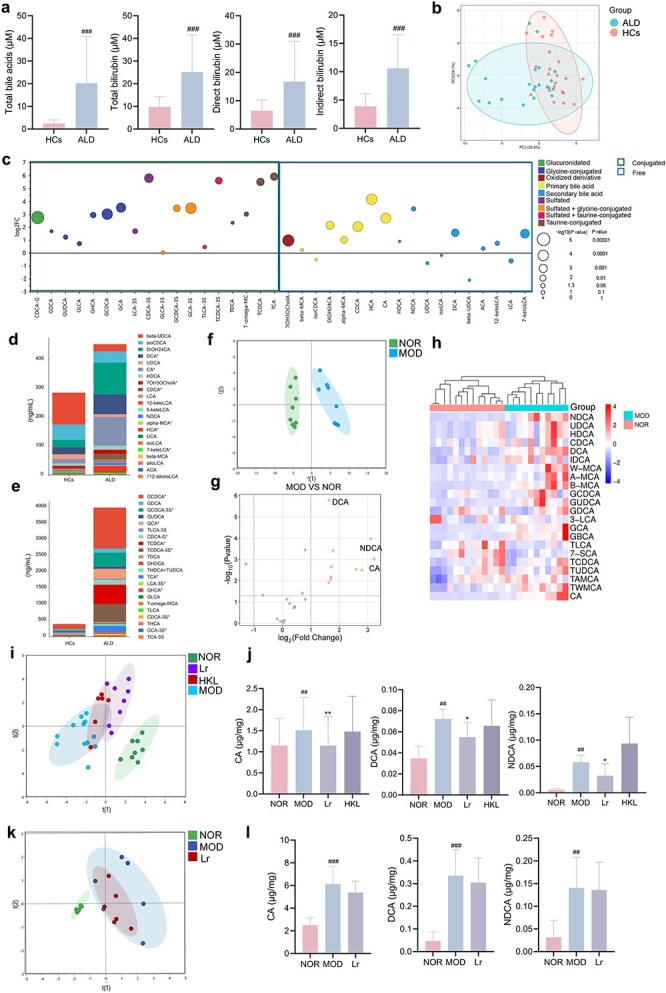
Excessive alcohol consumption disrupts bile acid metabolism both in ALD patients and mice, whereas *L. reuteri* demonstrates therapeutic potential in restoring homeostasis; (a) the levels of TBA, total bilirubin (TBIL), direct bilirubin (DBIL), and indirect bilirubin (IBIL) of HCs and ALD patients; (b) PCA score plot of bile acids in serum of HCs and ALD patients; (c) scatter plot of bile acids in serum of HCs and ALD patients; (d) free bile acids in serum of HCs and ALD patients; (e) conjugated bile acids in serum of HCs and ALD patients; (f) PLS-DA score plot of bile acids in liver of mice; (g) volcano plot of bile acids in liver of mice; (h) heatmap analysis of differentially bile acids in mice; (i) PLS-DA score plot of the bile acids in mice liver from *L. reuteri* supplementation groups in WT mice; (j) bar chart of liver bile acids in each group of WT mice (*n* = 8); (k) PLS-DA score plot of the bile acids in mice liver from *L. reuteri* supplementation groups in *Fxr*^−/−^ mice; (l) bar chart of liver bile acids in each group of *Fxr*^−/−^ mice (*n* = 8); HCs: healthy controls; ALD: ALD patients; compared with the HCs test, ^##^*P <* .01, ^###^*P <* .001; NOR, normal group; MOD: model group; Lr: ALD mice treated with *L. reuteri*; HKL: ALD mice treated with heat-killed *L. reuteri*; compared with the NOR, ^##^*P <* .01, ^###^*P <* .001; compared with the MOD, ^*^P < .05, ^**^P < .01.

As the core function of FXR is the regulation of bile acids, we systematically measured bile acid levels in both clinical patient samples and animal models. Clinical biomarker analysis revealed significantly elevated levels of total bile acids (TBA), total bilirubin, direct bilirubin, and indirect bilirubin in ALD patients compared to HCs (Fig. 6a), indicative of potential cholestatic pathology, which prompted our subsequent targeted bile acid metabolomics profiling of plasma samples to precisely quantify individual bile acid species and elucidate their metabolic perturbation. The PCA plot revealed distinct bile acid profiling between HCs and ALD, indicating metabolic dysregulation in ALD patients (Fig. 6b). A total of 36 bile acids were quantitatively profiled in human serum samples. A decrease in levels of only five bile acids, such as ursodeoxycholic acid and β-ursodeoxycholic acid, was observed in ALD patients, alongside elevated concentrations of 31 bile acids, such as CA, DCA, and taurocholic acid, in ALD patients (Fig. 6c and Supplementary Table S9). To more comprehensively visualize the bile acid profile alterations, we employed stacked bar plots to demonstrate the relative abundances of bile acids in plasma samples. Analysis of conjugated and free bile acids revealed that both types accumulated in ALD patients, with conjugated bile acids exhibiting more severe accumulation. Statistical analysis identified 19 bile acids with significant intergroup differences (*P <* .05), all demonstrating elevated concentrations in ALD patients (Fig. 6d and e).

To determine whether the observed differences were independent of alcohol exposure, we performed ANCOVA with adjustment for alcohol consumption, duration of alcohol use, and disease stage. After adjustment for these confounders, the previously significant between-group differences in bile acid metabolites such as CA, DCA, and 23-nordeoxycholic acid (NDCA) and gut microbiota (*Lactobacillus* and *L. reuteri*) were no longer statistically significant (Supplementary Table S10). These results indicate that the observed alterations in bile acids and gut microbiota are primarily attributable to alcohol exposure and disease progression, which is fully consistent with the established pathogenesis of ALD.

To establish a clinical association network integrating gut microbiota, bile acid metabolites, and disease parameters, Spearman correlation analysis was performed. The correlation heatmap showed that the abundance of *Lactobacillus* and *L. reuteri* was significantly negatively correlated with ALD, disease stage, alcohol consumption, bile acid levels, and several clinical parameters. In contrast, bile acids were significantly positively correlated with alcohol consumption, ALD, and multiple liver function indicators, particularly CA (Supplementary Figure S7A). To further visualize the interconnections among these variables, a correlation network graph was constructed using only variable pairs with statistically significant correlations (*P <* .05) (Supplementary Figure S7B). Collectively, these two complementary visualization approaches establish a robust “microbiota-metabolite-disease” association network, providing clinical evidence supporting the gut–liver axis in ALD pathogenesis.

The PCA score plots of bile acid-targeted metabolomics in mouse liver tissues showed that the samples within each group clustered well, and there was a clear separation trend between the NOR and MOD groups, indicating abnormal metabolic profiles in ALD mice (Fig. 6f). A total of 12 bile acids showed significant differences between the NOR and MOD groups (Supplementary Table S11). The volcano plot and heatmap analysis of the differential bile acids revealed that, compared to the NOR group, the MOD group exhibited significantly increased levels of CA, DCA, and NDCA (*P <* .05) (Fig. 6g and h).

Supplementation with *L. reuteri* modulated the bile acid metabolic profile, significantly reversing the levels of CA, DCA, and NDCA (Fig. 6i and j)*.* These results indicate that ALD induces gut dysbiosis and bile acid abnormalities, both of which are reversed by *L. reuteri* supplementation. We next analyzed bile acid profiles in *Fxr*^−/−^ mice supplemented with *L. reuteri* and observed that the dysregulated bile acid patterns failed to normalize, with no significant reduction in the levels of CA, DCA, and NDCA (Fig. 6k and l). Collectively, these results demonstrate that the ameliorative effects of *L. reuteri* on ALD-associated bile acid dysregulation are strictly dependent on FXR-mediated pathways.

### Bile acid supplementation induced hepatotoxicity in both liver cells and mice

**Figure 7:**
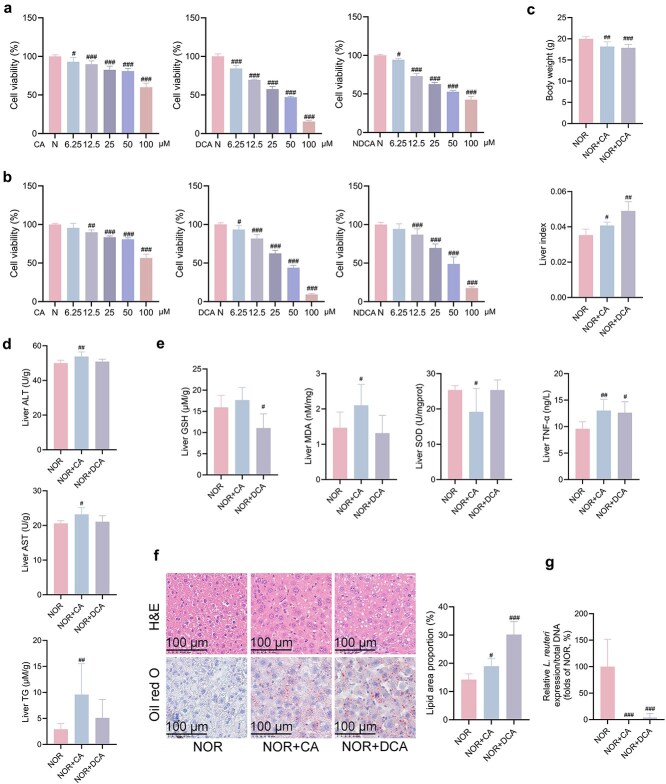
Bile acid supplementation induces hepatotoxicity and reduces *L. reuteri* abundance; (a) cell viability of HepG2 cell treated with bile acids (*n* = 4); (b) cell viability of L02 cell treated with bile acids (*n* = 4); (c) body weight and liver index in each group (*n* = 12); (d) the levels of ALT, AST, and TG in each group (*n* = 8); (e) the levels of GSH, MDA, SOD, and TNF-α in each group (*n* = 8); (f) H&E staining and oil red O staining of liver in each group (*n* = 3), scale bar = 100 μm; (g) relative abundance of *L. reuteri* in feces; NOR: normal group; NOR + CA: normal mice treated with CA; NOR + DCA: normal mice treated with DCA; compared with the NOR, ^#^*P <* .05, ^##^*P <* .01, ^###^*P <* .001.

The above study observed significantly elevated levels of CA, DCA, and NDCA in both ALD patients and mouse models, all of which were effectively modulated by *L. reuteri*. Whereas the hepatotoxicity of DCA has been documented, the potential liver injury effects of CA and NDCA remain largely unclear [42, 43]. To systematically evaluate the hepatotoxic potential of these bile acids and their potential roles in ALD pathogenesis, we conducted both *in vitro* and *in vivo* hepatotoxicity evaluation of bile acids based on the differential bile acids identified in ALD mice and the regulatory effects of *L. reuteri*. The toxicity of CA, DCA, and NDCA on HepG2 and L02 cells was evaluated by MTT assay (Fig. 7a and b). CA, DCA, and NDCA all reduced the viability of HepG2 and L02 cells.

Given the pronounced hepatotoxicity observed *in vitro* for both CA and DCA, it is imperative to undertake a comprehensive *in vivo* hepatotoxicity assessment of these two bile acids. After CA and DCA were administered to normal mice, a significant decrease in body weight was observed, accompanied by an increase in liver index (Fig. 7c). In the NOR + CA group, levels of ALT, AST, TG, MDA, and TNF-α were significantly increased, whereas SOD was significantly decreased (Fig. 7d and e). In the NOR + DCA group, TNF-α level was significantly increased whereas GSH level was significantly decreased (Fig. 7d and e). The histological analysis revealed elevated levels of lipid accumulation and hepatic vacuolation in both NOR + CA and NOR + DCA groups (Fig. 7f). Furthermore, both CA and DCA significantly reduced the fecal abundance of *L. reuteri* compared with the NOR group (Fig. 7g).

Having established that exogenous CA and DCA are hepatotoxic in healthy mice, we next asked whether these bile acids could exacerbate pre-existing ALD pathology. To address this, we administered CA and DCA to ALD mice, simulating the additional burden of elevated bile acids beyond the endogenous dysregulation already present in the disease. Both treatments showed no ameliorative effects and instead aggravated ALD symptoms. Compared with the MOD group, CA and DCA supplementation did not significantly reduce body weight but increased the liver index (Supplementary Figure S8A). DCA supplementation led to significantly higher plasma ALT levels than those in the MOD group (Supplementary Figure S8B). CA supplementation resulted in elevated MDA levels compared with the MOD group (Supplementary Figure S8C). Moreover, both CA and DCA supplementation caused significantly higher levels of inflammatory cytokines than those in the MOD group (Supplementary Figure S8D). Oil Red O staining of liver sections revealed that DCA supplementation induced more severe lipid accumulation (Supplementary Figure S8E). Collectively, these results indicate CA and DCA not only are directly hepatotoxic but also can worsen ALD-induced liver injury, reinforcing the pathogenic role of bile acid accumulation in ALD progression.

FXR, as a central regulator of bile acid homeostasis, forms a bidirectional feedback loop with bile acids—particularly DCA and other bile acids can promote their own clearance by activating FXR [21]. To systematically elucidate the direct hepatotoxic effects of these bile acids independent of FXR-mediated regulation, we administered CA and DCA to *Fxr^−/−^* mice. Our results demonstrated that both CA and DCA retained their hepatotoxic effects in the absence of FXR, with DCA exhibiting particularly pronounced toxicity (Supplementary Figure S9). DCA has been reported to serve as a more potent FXR activator than CA [44]. Thus, in WT mice, DCA-mediated FXR activation promotes bile acid clearance and reduces hepatic injury, thereby providing mechanistic support for *L. reuteri*-mediated amelioration of ALD through FXR activation and subsequent cholestasis relief. Collectively, these findings highlight the critical role of FXR-mediated bile acid metabolism and clearance mechanisms in maintaining liver health.

### Echinacoside enriched *Lactobacillus reuteri* to improve alcoholic liver disease

**Figure 8:**
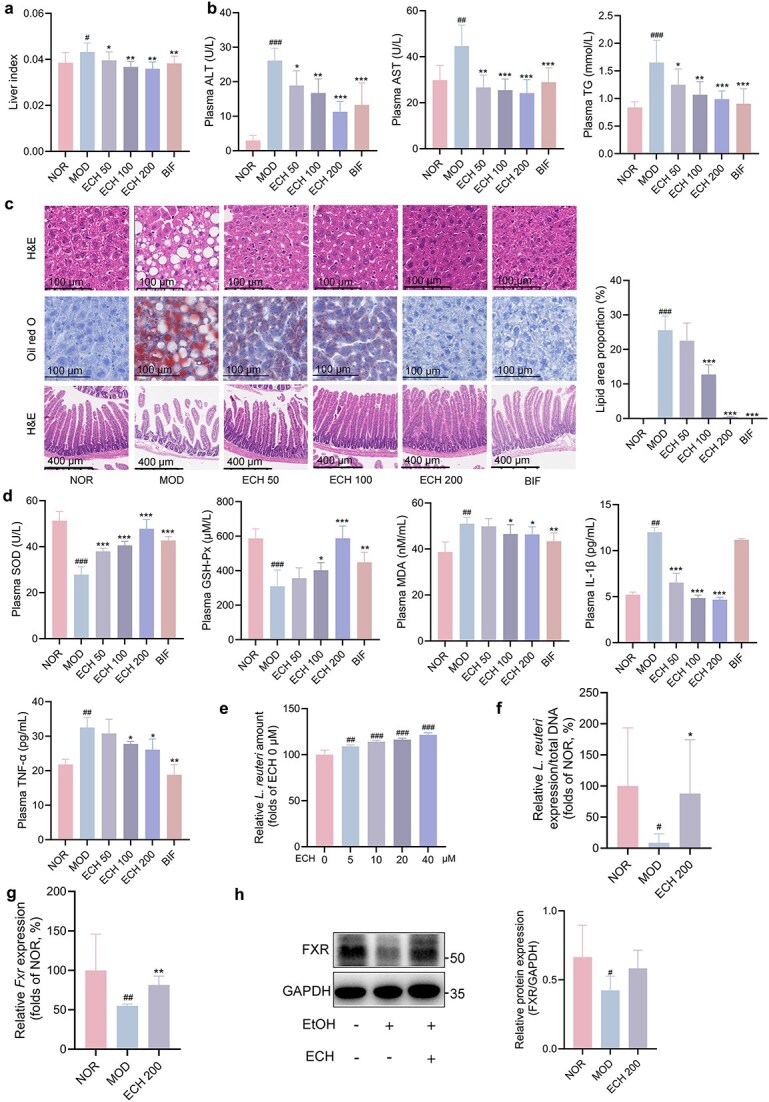
ECH ameliorates ALD by modulating the *L. reuteri*-mediated FXR activation; (a) liver index in each group (*n* = 12); (b) ALT, AST, and TG in each group (*n* = 8); (c) H&E staining of liver and small intestine tissues, and oil red O staining in each group (*n* = 3); (d) SOD, GSH-Px, MDA, IL-1β, and TNF-α in each group (*n* = 8); (e) relative amount of *L. reuteri* (*n* = 3); (f) relative abundance of *L. reuteri* in fecal samples (*n* = 6); (g) the relative mRNA expression levels of *Fxr* in mice liver (*n* = 6); (h) the protein expression levels of FXR in mice liver (*n* = 4); NOR: normal group; MOD: model group; ECH 50: ALD mice treated with ECH (50 mg/kg); ECH 100: ALD mice treated with ECH (100 mg/kg); ECH 200: ALD mice treated with ECH (200 mg/kg); compared with the NOR or 0 μM, ^#^*P <* .05, ^##^*P <* .01, ^###^*P <* .001; compared with the MOD, ^*^P < .05, ^**^P < .01, and ^***^P < .001.

ECH is the most abundant bioactive ingredient in Cistanches Herba, *E. angustifolia*, and *L. lucidum* [31]. ECH has demonstrated hepatoprotective effects in acute ALD; however, its efficacy in chronic ALD progression remains uninvestigated [30, 34]. The underlying mechanisms of ECH are still unclear, and no studies have yet investigated its prebiotic properties or related mechanisms. Moreover, ECH was reported to alleviate type 2 diabetes mellitus through inhibiting hepatic gluconeogenesis via gut bacterial-fungal trans-kingdom network, with the abundances of *Lactobacillus* increased [45], which indicates that ECH may have the potential to promote the growth of *L. reuteri* to alleviate ALD*.* In the present study, we found that ECH could improve liver injury in NIAAA model, including liver index, AST, ALT, TG, oxidative stress biomarkers, pro-inflammatory factor levels, and histology (Fig. 8a–d). *In vitro* analysis revealed that ECH could effectively promote the growth of *L. reuteri* (Fig. 8e). ECH significantly increased *L. reuteri* yield in nutrient-rich GAM medium (Supplementary Figure S10A) and supported modest growth in carbon-free GAM medium (Supplementary Figure S10B), indicating that it promotes bacterial growth as both a signaling molecule and marginal carbon source. Moreover, ECH administration significantly increased the abundance of *L. reuteri* in the feces of ALD mice (Fig. 8f).

The Western blot and qRT-PCR experiments showed that ECH could significantly activate the expression of FXR in the liver (Fig. 8g and h and Supplementary Figure S5D). The results of bile acid targeted metabolomics showed that ECH reduced the levels of bile acids in liver, especially CA, DCA, and NDCA, indicating that ECH promoted the excretion of bile acids (Supplementary Figure S11). The above results indicate that ECH can improve ALD by promoting the growth of *L. reuteri* and regulating FXR and bile acid balance.

To evaluate the impact of ECH on gut microbiota, 16S rRNA gene sequencing was performed. PCoA analysis showed a clear separation between the NOR and MOD groups, whereas the ECH 200 group clustered closer to the NOR group (Supplementary Figure S12A), indicating that ECH partially reversed alcohol-induced gut microbiota dysbiosis. At the phylum level, ECH restored the relative abundance of Bacteroidetes and reduced alcohol-enriched Firmicutes and Proteobacteria (Supplementary Figure S12B). At the genus level (Supplementary Fig. S12C), beyond the recovery of *Lactobacillus*, ECH significantly enriched beneficial bacteria such as *Muribaculaceae* and *Lachnospiraceae_NK4A136_group*. These findings reveal that ECH reconfigures the alcohol disrupted gut microbiota toward a healthier community state, characterized by coordinated shifts at both phylum and genus levels, and imply a network-based regulatory mechanism.

## Discussion

At present, research on the pathological mechanism of ALD mainly focuses on oxidative stress, inflammatory response, and lipid metabolism disorders caused by ethanol and acetaldehyde [46]. With the continuous deepening of research on gut microbiota, the study of ALD mechanism and the exploration of therapeutic targets based on gut microbiota have gradually become hot topics [47]. Here, we found changes in the gut microbiota of ALD patients and model mice, confirming the therapeutic potential of functional differentially expressed *L. reuteri* in clinical ALD. Through mechanism research, it was found that *L. reuteri* could inhibit cholestasis and improve ALD by activating FXR target. Furthermore, a bioactive natural product, ECH, was disclosed to act as a prebiotic to improve ALD by promoting the growth of *L. reuteri*, and targeting FXR to ameliorate ALD.

Recent advances highlight the therapeutic potential of probiotics and post-biotics in ALD. Meta-analyses of randomized controlled trials indicate that probiotic supplementation significantly improves liver function and modulates gut microbiota in ALD patients, increasing beneficial bacteria such as *Bifidobacterium* and *Lactobacillus* [8, 9]. Specifically, *Bifidobacterium lactis* Probio-M8 fermented milk has been shown to alleviate alcohol-induced liver injury by maintaining gut microbiota stability [48]. Moreover, post-biotics derived from *Saccharomyces cerevisiae* and *Lactobacillus plantarum* alleviate ALD by restoring intestinal barrier integrity and promoting bile acid metabolism [49]. Additionally, the natural small molecule cyanidin-3-*O*-glucoside alleviates ALD by increasing the abundance of *Muribaculaceae* [50]. Collectively, these studies demonstrate the efficacy of probiotics, post-biotics, and natural products in the management of ALD. However, the specific mechanisms and molecular targets remain incompletely defined.

*Lactobacillus reuteri* belongs to the genus of *Lactobacillus* and is beneficial for intestinal barrier function. Previous studies have shown that *L. reuteri* might reduce oxidative stress and inflammatory response in mice with alcoholic steatohepatitis and improve liver lipid accumulation in ALD mice by regulating the FXR signaling pathway [21, 26, 51]. Nevertheless, the mechanistic basis underlying the therapeutic effects of *L. reuteri* in ALD remains poorly characterized, and the potential role of FXR as a critical molecular target in this process has yet to be investigated. Here, we first validated this hypothesis in liver cells, demonstrating FXR activation by the culture supernatant of *L. reuteri*, with consistent results observed in animal livers. Our research results further showed that ALD leads to the inhibition of FXR and that the knock-out of Fxr exacerbates ALD. *L. reuteri* improved ALD by activating FXR; however, it significantly weakened its effect on improving liver indicators in *Fxr*^−/−^ mice. This study provides solid evidence that *L. reuteri* exerts its hepatoprotective effects against ALD through FXR-dependent mechanisms. This sheds light on the therapeutic role of *L. reuteri* and the pivotal involvement of FXR in ALD pathogenesis.

FXR is a bile acid receptor, which plays a crucial role in maintaining bile acid homeostasis and regulating metabolic processes [12]. There exists a complex bidirectional regulatory relationship between FXR and bile acids in ALD, where they are mutually dependent and influence each other [21, 22]. However, the precise mechanisms of *L. reuteri* on FXR and bile acids have not yet been fully elucidated. To address this, we employed bile acid-targeted metabolomics to analyze the regulatory effects of *L. reuteri* on bile acid profiles in both WT and *Fxr*^−/−^ ALD models. Our results demonstrated that *L. reuteri* effectively ameliorated bile acid dysregulation in WT mice but failed to restore bile acid homeostasis in *Fxr*^−/−^ animals. These findings indicate that *L. reuteri* exerts its therapeutic effects on ALD primarily through FXR-dependent mechanisms to alleviate cholestasis.

FXR exhibits context-dependent and cell-type-specific roles in liver diseases. Several studies have reported that FXR activation exerts anti-fibrotic effects in hepatic stellate cells by inhibiting their activation and promoting quiescence [52, 53]. Conversely, other studies have suggested that FXR inhibition may ameliorate liver fibrosis [54]. This apparent discrepancy can be reconciled by considering differences in disease etiology, pathological stage, and the dominant pathogenic mechanisms at play. In cholestatic or toxic injury models where bile acid accumulation is a primary driver of pathology, FXR inhibition may reduce bile acid synthesis and toxicity, thereby conferring benefit. By contrast, in ALD, where gut-derived endotoxins, intestinal barrier disruption, and bile acid dysregulation are key pathogenic factors, FXR activation in both the intestine and liver reinforces intestinal barrier integrity, suppresses hepatic inflammation, and restores bile acid homeostasis, collectively conferring hepatoprotection [55, 56]. Therefore, although the role of FXR in liver fibrosis may vary across disease contexts, our findings support that FXR activation represents a beneficial strategy specifically in ALD.

To elucidate the mechanism by which *L. reuteri* activates FXR, we analyzed its culture supernatant and gut microbiota composition. Molecular docking revealed that several metabolites enriched in *L. reuteri* culture supernatant stably bound to the FXR ligand-binding pocket. Structural studies have shown that FXR activation involves cation-π interactions between the indole ring of histidine on H10/H11 and aromatic side chains on H12, stabilizing the receptor in its bioactive conformation [40]. Additionally, 16S rRNA gene sequencing revealed that *L. reuteri* supplementation significantly reshaped the gut microbiota composition, with increased abundance of *L. johnsonii* and *F. rodentium. L. johnsonii* has been shown to regulate bile acid metabolism and modulate the intestinal FXR-FGF15-CYP7A1 axis [57], whereas *F. rodentium*-derived metabolites have been reported to activate FXR via the gut microbiota–metabolite axis [58]. Collectively, these findings suggest that *L. reuteri* activates FXR through both metabolite-driven and microbiota-mediated mechanisms, thereby regulating bile acid metabolism and hepatic function.

Cholestasis is a common physiological feature of ALD patients, and excessive elevation of bile acid levels is significantly positively correlated with alcoholic hepatitis scores, with serum bile acid levels continuously increasing as ALD progresses [59, 60]. Previous studies have shown that serum levels of TBA and conjugated bile acids, such as taurochenodeoxycholic acid, are significantly elevated in ALD patients and positively correlated with disease severity [61]. The targeted measurement of bile acids in ALD mice showed that the levels of free bile acids, such as CA, DCA, and muricholic acid, were significantly increased [62]. Our study extends these observations by revealing that ALD patients display not only cholestasis but also concurrent increases in both conjugated and unconjugated bile acids, with the unconjugated forms (particularly CA, DCA, and NDCA) demonstrating more pronounced hepatotoxic effects. These findings significantly expand our understanding of bile acid metabolic disturbances in ALD pathogenesis. Our comprehensive bile acid profiling in human serum and mice liver, combined with *in vitro* and *in vivo* toxicity assays in mice, provides robust evidence for the differential hepatotoxicity of specific bile acid subtypes.

Except for the present discovery of the role of *L. reuteri* in ameliorating bile acid dysregulation through FXR, previous studies have demonstrated that *L. reuteri* ameliorated chronic acrylamide-induced glucose metabolic disorders through modulation of bile acid metabolism [63]. Additionally, *L. reuteri* supplementation was also found to significantly reduce levels of DCA under LPS stimulation [64]. Extending these findings, our study revealed that *L. reuteri* modulated the levels of CA and NDCA in ALD patients and mice. Using normal WT and *Fxr*^−/−^ mice, we further elucidated the hepatotoxicity of DCA and CA. Additionally, supplementation with bile acids was found to reduce *L. reuteri* abundance in mouse intestine, providing further evidence for the bidirectional regulatory relationship between bile acids and *L. reuteri*.

Growing evidence has identified various natural product compounds or extracts as promising prebiotic candidates for ALD therapy [65, 66]. For instance, the total glycosides from *C. tubulosa* have demonstrated therapeutic efficacy against ALD through gut microbiota modulation [67]. ECH, the most abundant compound in the total glycosides from *C. tubulosa*, was also reported to increase the abundances of *Lactobacillus* in type 2 diabetes mice [45]. Here, we found that ECH had a liver protective effect against ALD and further disclosed its mechanism as a prebiotic for *L. reuteri* to activate FXR. ECH was found to be able to significantly increase the abundance of *L. reuteri* both *in vitro* and *in vivo*, and activated FXR expression level and reduced the concentrations of bile acids, such as CA and DCA, in the liver, thereby exerting an anti-ALD effect. These results suggest that ECH may be a promising prebiotic for the treatment of ALD.

In conclusion, this study clinically identified and validated the deficiency of *L. reuteri* and the elevated levels of bile acids in ALD patients, and systematically reveals the mechanism by which *L. reuteri* regulates bile acid homeostasis by targeting FXR, thereby exerting liver protective effects. Furthermore, the natural product ECH exhibited a favorable ability to resist ALD by promoting the growth of *L. reuteri* and activating FXR expression. Our study presents the gut microbe *L. reuteri* as a probiotic and ECH as a prebiotic agent for the treatment of ALD, and further validates the crucial function of bile acid metabolism orchestrated by the gut microbiota in maintaining a healthy liver. These findings provide a new theoretical basis and therapeutic means for ALD. Our findings illustrate how a single microbial strain can orchestrate ecosystem-level changes in the gut microbiota and bile acid pool, thereby influencing host physiology. This underscores the importance of understanding microbial systems as integrated functional units rather than isolated entities.

## Supplementary Material

Supplementary_materials_-_final_-_0612_wrag153

## Data Availability

The 16S rRNA genetic sequencing datasets have been deposited in the National Center for Biotechnology Information (Accession number: PRJNA1302439, https://www.ncbi.nlm.nih.gov/sra/?term=PRJNA1302439). The metabolomics data were deposited in CNCB (https://ngdc.cncb.ac.cn/omix). The project number is PRJCA047206 (https://ngdc.cncb.ac.cn/bioproject/browse/PRJCA047206).
