# Supplementary material for: Lactobacillus reuteri targets farnesoid X receptor-dependent bile acid homeostasis to ameliorate alcoholic liver disease
Source: ISME J. 2026 Jun 16;20(1):wrag153. doi: 10.1093/ismejo/wrag153 (PMC13471275; doi:10.1093/ismejo/wrag153)
Supplement: Supplementary file 1 [file supplementary_materials_-_final_-_0612_wrag153.docx]

**Supplementary materials**

**Content list：**

**1.1** Animal modeling and drug administration

**1.2** Immunofluorescence analysis

**1.3** 16S rRNA gene sequencing

**1.4** Targeted metabolomics (T700)

**1.5** Bile acid-targeted metabolomics of human serum

**1.6** Bile acid-targeted metabolomics of mouse liver

**1.7** Bacterial growth curve assay

**1.8** Correlation analysis and network construction

**1.9** Analysis of covariance (ANCOVA)

**Table S1** The methodological approach for bile acid targeted metabolomics of serum samples

**Table S2** The mobile phase gradient elution condition

**Table S3** The bile acid standards and internal standards of bile acid targeted metabolomics

**Table S4** The mass spectrum parameters of bile acids

**Table S5** The primer sequences of qRT-PCR

**Table S6** Baseline of healthy controls (HCs) and ALD patients

**Table S7** L. reuteri-enriched metabolites identified by targeted metabolomics (T700).

**Table S8** Screening of potential FXR agonists from L. reuteri-enriched metabolites by molecular docking

**Table S9** Statistical analysis of serum bile acid levels of HCs and ALD

**Table S10** Adjusted *P* value of key differential metabolites and gut microbiota between HCs and ALD group using ANCOVA

**Table S11** Differential bile acid in liver of NOR and MOD mice

**Figure S1** Alterations in gut microbiota and barrier function in ALD mice. (A) Alpha diversity of fecal samples between NOR and MOD mice (*n* = 8). (B) Principal coordinates analysis (PcoA) plot of fecal samples between NOR and MOD mice. (C) Circos plot of fecal samples between NOR and MOD mice. (D) H&E staining of small intestine tissues in NOR and MOD mice. (E) Plasma lipopolysaccharide (LPS) levels in NOR and MOD mice (*n* = 8). (F) Immunohistochemical staining of Occludin in small intestine tissues in NOR and MOD mice. NOR: normal group; MOD: model group. Compared with the NOR, ^##^*P <* 0.01, ^###^*P <* 0.001.

**Fig. S2** *L. reuteri* administration effectively restored the impaired intestinal barrier in ALD mice. (A) Immunofluorescence staining of Occludin in intestinal tissues. (scale bar = 500 µm, *n* = 3). (B) Immunofluorescence staining of ZO-1 in intestinal tissues. (scale bar = 500 µm, *n* = 3). (C) Immunofluorescence staining of Claudin-1 in intestinal tissues. (scale bar = 500 µm, *n* = 3). NOR: normal group; MOD: model group; Lr: ALD mice treated with *L. reuteri*. Compared with NOR, ^#^*P <* 0.05, ^##^*P <* 0.01; compared with MOD, **^*^***P <* 0.05, **^**^***P <* 0.01.

**Fig. S3** Metabolomic profiling of fecal contents following *L. reuteri* administration using the T700 platform. (A) Partial least squares discriminant analysis (PLS-DA) score plot showing the separation among different groups. (B) Permutation test (200 times) for model validation. (C) Hierarchical tree map illustrating the structural classes of differential metabolites between the NOR and MOD groups. (D) Hierarchical tree map illustrating the structural classes of differential metabolites between the MOD and Lr groups. NOR: normal group; MOD: model group; Lr: ALD mice treated with *L. reuteri*.

**Fig. S4** Immunofluorescence staining of GPCR43 and AHR in intestinal tissues. (scale bar = 500 µm，*n* = 3). NOR: normal group; MOD: model group; Lr: ALD mice treated with *L. reuteri*. Compared with MOD, ^*^*P <* 0.05.

**Fig. S5** Western blot replications of cell and mice liver. (A) Western blot replications of HepG2 cell (*n* = 4). (B) Western blot replications of L02 cell (*n* = 4). (C) Western blot replications in mice liver of *L. reuteri* (*n* = 4). (D) Western blot replications in mice liver of ECH (*n* = 4). Lr: ALD mice or cell treated with *L. reuteri*; HKL: ALD mice or cell treated with heat-killed *L. reuteri*. ECH: ALD mice treated with ECH (200 mg/kg).

**Fig. S6**Analysis of potential metabolites enriched by *Lactobacillus reuteri* (*L. reuteri*) using T700. (A) Principal component analysis (PCA) score plot between groups. (B) Heatmap of all detected compounds across samples. (C) Volcano plot of all detected compounds. (D) Hierarchical tree map of the structural classes of potential metabolites enriched by *L. reuteri.* (E) Sankey and heatmap diagram of potential metabolites enriched by *L. reuteri.* Lr: the supernatant of *L. reuteri*；HKL: the supernatant of heat-killed *L. reuteri.*

**Fig. S7** Association analysis of gut microbiota, metabolites, and disease indices in human samples. (A) Spearman correlation heatmap showing pairwise relationships among differential metabolites, gut bacterial genera, and baseline clinical parameters. Red indicates positive correlation; blue indicates negative correlation. Color intensity represents correlation strength. ^*^*P <* 0.05, and ^**^*P <* 0.01. (B) Correlation network diagram constructed using only variables with significant correlations. Nodes represent individual variables; edges represent significant correlations; edge thickness reflects correlation strength; yellow edges indicate positive correlations; grey edges indicate negative correlations. Note: ALD: alcoholic liver disease; TP: total protein; TBA: total bile acids; CHE: cholinesterase; ALB: albumin; GLB: globulin; AG: albumin/globulin ratio; TBIL: total bilirubin; DBIL: direct bilirubin; IBIL: indirect bilirubin; ALT: alanine aminotransferase; AST: aspartate aminotransferase; GGT: gamma-glutamyl transferase; TC: total cholesterol; TG: triglycerides; LDLC: low-density lipoprotein cholesterol; HDLC: high-density lipoprotein cholesterol; GLU: Glucose.

**Fig. S8** Impact of bile acid administration on physiological parameters in ALD mice. (A) Body weight and liver index in each group (*n* = 8). (B) ALT, AST, and TG levels in each group (*n* = 8). (C) GSH-Px, SOD, and MDA levels in each group (*n* = 8). (D) IL-1β and TNF-α levels in each group (*n* = 8). (E) H&E staining of liver and small intestine tissues, and Oil red O staining in each group (*n* = 3), scale bar = 100 µm. NOR: normal group; MOD: model group; CA: ALD mice treated with CA; DCA: ALD mice treated with DCA. Compared with the NOR, ^#^*P <* 0.05, ^##^*P <* 0.01, ^###^*P <* 0.001, ^####^*P <* 0.0001; compared with the MOD, ^*^*P <* 0.05, ^***^*P <* 0.001.

**Fig. S9** Impact of bile acid administration on physiological parameters in *Fxr*^-/-^normal mice. (A) Body weight, liver index, ALT, AST, GSH-Px, MDA, TNF-α, IL-1β levels in each group (*n* = 10). (B) H&E staining of small intestine tissues and Oil red O staining of liver (*n* = 3), scale bar =100 µm. NOR: normal group; NOR+CA: normal mice treated with CA; NOR+DCA: normal mice treated with DCA. Compared with the NOR, ^#^*P <* 0.05, ^##^*P <* 0.01, ^###^*P <* 0.001.

**Fig. S10** Echinacoside (ECH) promotes *L. reuteri* growth. (A) Growth curves of *L. reuteri* in standard GAM medium. (B) Growth curves of *L. reuteri* in modified GAM broth without glucose and soluble starch. Values with different lowercase letters indicate statistically significant differences among groups at the level of *P* < 0.05. Samples sharing at least one common lowercase letter have no significant difference, whereas those with completely different lowercase letters are significantly different.

**Fig. S11** Regulatory effects of ECH on bile acid metabolism in ALD mice. Bar chart of liver bile acids in each group (*n* = 8). NOR: normal group; MOD: model group; ECH 50: ALD mice treated with ECH (50 mg/kg); ECH 100: ALD mice treated with ECH (100 mg/kg); ECH 200: ALD mice treated with ECH (200 mg/kg). Compared with the NOR, ^#^*P <* 0.05, ^##^*P <* 0.01, ^###^*P <* 0.001; compared with the MOD, **^*^***P <* 0.05, **^**^***P <* 0.01, and **^***^***P <* 0.001.

**Fig S12** Regulatory effects of ECH on gut microbiota in ALD mice. (A) Principal coordinates analysis (PCoA) plot of fecal samples. (C) Bubble plot of the genus level. NOR: normal group; MOD: model group; ECH 200: ALD mice treated with ECH (200 mg/kg).

**1.1 Animal modeling and drug administration**

Ethanol Feeding Regimen: All animals were initially acclimated to a control liquid diet for 5 days. Starting on day 6, only the normal (NOR) group remained on the control diet. The other five groups, designated for model induction, were switched to an ethanol Lieber-DeCarli liquid diet containing 5% (*v/v*) ethanol. Daily monitoring of body weight and food consumption was performed to maintain matched caloric intake.

Ethanol Binge and Pharmacological Intervention: On the morning of day 16, after an 8-hour fast, mice in the ethanol-fed groups were orally gavaged with a single binge dose of ethanol (31.5% *v/v*, 5 g/kg). The NOR group received an isocaloric maltodextrin solution (45% *w/v*) at the same volume. Throughout the 25-day ethanol feeding period, the respective intervention groups received daily oral administrations of bacterial suspensions or active compounds. The NOR and model (MOD) groups received a comparable volume of vehicle (water) daily.

**1.2 Immunofluorescence analysis**

Intestinal sections were deparaffinized, rehydrated, and subjected to antigen retrieval in sodium citrate buffer (pH 6.0). After blocking with 5% BSA containing 0.3% Triton X-100, sections were incubated with primary antibodies against ZO-1, Occludin, Claudin-1, GPR43, or AhR overnight at 4°C. Following washing, corresponding fluorophore-conjugated secondary antibodies were applied for 1 h at room temperature. Nuclei were counterstained with DAPI. Images were captured using a confocal microscope, and fluorescence intensity was quantified using ImageJ software (NIH, USA).

**1.3 16S rRNA gene sequencing**

Genomic DNA was extracted from intestinal microbiota samples using the E.Z.N.A.NA Kit (Omega Bio-Tek, Norcross, GA, USA), with subsequent dilution to a working concentration of 1 ng/μL using molecular-grade sterile water. The hypervariable V3-V4 regions of bacterial 16S rRNA genes were amplified using barcoded primers 341F (5'-ACTCCTACGGGAGGCAGCAG-3') and 806R (5'-GGACTACHVGGGTATCTAAT-3'). High-throughput sequencing analysis of bacterial rRNA genes was performed by a Hiseq 2500 System (Illumina) provided by Biotree Co. Ltd. (Shanghai, China).

**1.4 Targeted metabolomics (T700)**

The targeted metabolomics assay was performed using the Maiwei T700 method developed by Wuhan Maiwei Metabolism Biotechnology Co., Ltd., and was applied to both fecal content and bacterial supernatant samples. Specifically, 50 mg of sample was accurately weighed into a 2 mL tube, followed by the addition of 500 µL of pre-chilled (-20°C) 70% methanol in water. The mixture was vortexed for 3 min and centrifuged at 12,000 rpm for 10 min at 4°C. A 300 µL aliquot of the supernatant was transferred to a new tube, incubated at -20°C for 30 min, and centrifuged again under the same conditions. The resulting supernatant (200 µL) was passed through a protein precipitation plate. For short-chain fatty acid (SCFA) analysis, 30 µL of the extract was sequentially mixed with 5 µL of 200 mM 3-NPH and 5 µL of 150 mM EDC, derivatized at 30°C for 60 min, and quenched with 1460 µL of pre-chilled acetonitrile. LC-MS/MS analysis was performed on an ExionLC AD UPLC system coupled with a QTRAP 6500+ mass spectrometer, using a Waters ACQUITY UPLC HSS T3 C18 column (1.8 µm, 2.1 × 100 mm). The mobile phases were (A) water with 0.05% formic acid and (B) acetonitrile with 0.05% formic acid, at a flow rate of 0.35 mL/min, column temperature of 40°C, and injection volume of 2 µL. The gradient program was: 0–8 min, 5%–95% B; 8–12.5 min, 95% B; 12.6–15 min, 5% B. Mass spectrometry was conducted using an electrospray ionization (ESI) source at 550°C, with ion spray voltages of 5500 V (positive mode) and -4500 V (negative mode), and curtain gas at 35 psi. Data acquisition were performed in multiple reaction monitoring (MRM) mode for targeted metabolite detection. Metabolites were identified using a standard compound database, and calibration curves were established using a series of standard solutions at concentrations ranging from 0.01 to 10,000 ng/mL. Data processing were carried out with Analyst 1.6.3 and MultiQuant 3.0.3 software, and metabolite concentrations in the samples were calculated by interpolating peak areas into the corresponding calibration curves.

**1.5 Bile acid-targeted metabolomics of human serum**

Aliquots of specimens were transferred to 2 mL centrifuge tubes, followed by precise addition of 400 μL pre-chilled methanol (–20°C). After vortex mixing for 60 s with two stainless steel beads, samples were homogenized at 55 Hz for 1 min using a tissue grinder (repeated twice). Subsequent ultrasonication was performed at room temperature for 30 min, followed by centrifugation at 12,000 rpm (4°C, 10 min). For analysis, 240 μL methanol was used initially, with 100 μL supernatant subsequently mixed with 200 μL water. All processed supernatants were diluted 20-fold with 30% methanol, filtered through 0.22 μm membranes, and transferred to autosampler vials.

The analytical platform comprised a liquid chromatography system (AB Sciex, USA) coupled with a triple quadrupole mass spectrometer (AB Sciex, USA). Separation was achieved on an ACQUITY UPLC BEH C_18_ column (2.1 × 100 mm, 1.7 μm; Waters, USA) maintained at 40°C. The mobile phase consisted of (A) 0.01% formic acid in water and (B) acetonitrile, delivered at 0.25 mL/min with the following gradient program: 0–4 min (25% B), 4–9 min (25–30% B), 9–14 min (30–36% B), 14–18 min (36–38% B), 18–24 min (38–50% B), 24–32 min (50–75% B), 32–33 min (75–90% B), and 33–35.5 min (90–25% B). Injection volume was 5 μL.

Electrospray ionization (ESI) was operated in negative ion mode with the following parameters: ion source temperature 500°C, ion spray voltage –4500 V, collision gas 6 psi, curtain gas 30 psi, nebulizer and auxiliary gases both at 50 psi. Data acquisition employed multiple reaction monitoring (MRM) scanning. The methodology validation was listed in Table S1.

**1.6 Bile acid-targeted metabolomics of mouse liver**

Liver samples were thawed gradually from -20°C to 0°C. Precisely 50 mg of tissue was homogenized in a 1:5 (*w/v*) ratio of ice-cold saline at 6.0 m/s for 40 s. From the homogenate, 100 μL was mixed with 1 mL of precooled extraction solvent (acetonitrile:isopropanol:water = 3:3:2, containing 0.02 μg/mL internal standard). After vortexing (3000 rpm, 10 min) and centrifugation (15,000 rpm, 10 min, 4°C), the supernatant was vacuum-dried. The extract was reconstituted in 100 μL of ice-cold methanol via sonication and vortexing, followed by a final centrifugation step. The resulting supernatant was subjected to LC-MS analysis for bile acid profiling.

An ACQUITY BEH C_18_ (2.1 × 100 mm, 1.7 μm) column was used for the separation of bile acids. The column temperature was set to 25°C, the flow rate was 0.2 mL/min, and the injection volume was 5 μL. Phase A and B was filled with 0.1% formic acid in water and acetonitrile, respectively. The mobile phase gradient elution conditions are shown in Table S2.

The MS conditions were as follows: ion mode: negative; acquisition mode: multiple reaction monitoring (MRM); dwell time: 20 msec; ion source temperature: 500°C; ion spray voltage: –4500 V; GS1: 50 psi; GS2: 50 psi; CUR: 35 psi; the collision gas: medium mode. The CE and DP of the bile acid standards and internal standards were displayed in Table S3. The mass spectrum parameters of the detected compounds are listed in Table S4.

**1.7 Bacterial growth curve assay**

*L. reuteri* was cultured anaerobically in GAM broth (Qingdao Hope Bio-Technology Co., Ltd., China) and in a modified GAM broth without glucose and soluble starch (Gifu Anaerobic Medium without Dextrose and Soluble Starch) at 37°C. The bacterial suspension was adjusted to an initial OD₆₀₀ of 0.05 and inoculated into 96‑well plates (100 μL per well). The plate was incubated anaerobically at 37°C, and OD₆₀₀ values were measured at 0, 2, 4, 6, 8, 12, 16, 20, 24, 36, and 48 h using a microplate reader (e.g., BioTek Epoch, USA). Each condition was performed in triplicate. Growth curves were plotted with time (h) as the x‑axis and OD₆₀₀ as the y‑axis.

**1.8 Correlation analysis and network construction**

Spearman correlation analysis was performed using SPSS 27.0 (IBM Corp., Armonk, NY, USA) to evaluate the relationships among *L. reuteri* abundance, bile acid metabolites (CA, DCA, and NDCA), clinical parameters (such as ALT, AST, and GGT), disease stage, and alcohol consumption. A correlation network was then constructed using only variable pairs with statistically significant correlations (*P <* 0.05). In the network, nodes represent individual variables, and edges represent significant correlations, with edge thickness reflecting correlation strength.

**1.9 Analysis of covariance (ANCOVA)**

To minimize the influence of potential confounders, ANCOVA was performed with adjustment for alcohol consumption, drinking duration, and disease stage. Group differences were considered statistically significant at *P <* 0.05. All analyses were conducted using SPSS (version 27.0, IBM Corp., Armonk, NY, USA).

**Table S1** The methodological approach for bile acid targeted metabolomics of serum samples

| No. | Compound name | Abbreviation | RT  (min) | Calibration_Curve | r | Linear_Range (ng/mL) | LOQ  (ng/mL) |
| --- | --- | --- | --- | --- | --- | --- | --- |
| 1 | Allolithocholic acid | alloLCA | 31.00 | y = 1.367e+04x + 1695 | 0.99 | 1.00 - 200.00 | 1.00 |
| 2 | Lithocholic acid | LCA | 33.02 | y = 6.832e+04x - 4.307e+04 | 0.99 | 1.25 - 200.00 | 1.25 |
| 3 | Isolithocholic acid | isoLCA | 31.45 | y = 2.608e+05x - 6.297e+04 | 0.99 | 0.50 - 400.00 | 0.50 |
| 4 | 23-Norcholic acid | NCA | 18.15 | y = 6239x + 1.67e+04 | 0.99 | 5.00 - 800.00 | 5.00 |
| 5 | 7-Ketolithocholic acid | 7-ketoLCA | 25.59 | y = 2.611e+04x - 1066 | 1.00 | 0.25 - 200.00 | 0.25 |
| 6 | 12-Ketolithocholic acid | 12-ketoLCA | 26.17 | y = 4007x - 300.7 | 1.00 | 1.25 - 1000.00 | 1.25 |
| 7 | 6-Ketolithocholic acid | 6-ketoLCA | 24.56 | y = 2209x + 277.7 | 0.99 | 1.00 - 50.00 | 1.00 |
| 8 | 3β-Ursodeoxycholic acid | beta-UDCA | 22.06 | y = 8.346e+04x - 667.1 | 0.99 | 0.50 - 400.00 | 0.50 |
| 9 | Deoxycholic acid | DCA | 28.69 | y = 2.354e+05x - 2.671e+04 | 1.00 | 0.50 - 400.00 | 0.50 |
| 10 | Chenodeoxycholic acid | CDCA | 28.12 | y = 1.493e+06x - 2.366e+04 | 0.99 | 0.50 - 100.00 | 0.50 |
| 11 | Ursodeoxycholic acid | UDCA | 23.48 | y = 7834x - 2.991 | 0.99 | 0.25 - 100.00 | 0.25 |
| 12 | Hyodeoxycholic acid | HDCA | 23.80 | y = 1.028e+05x - 2.095e+04 | 1.00 | 1.25 - 1000.00 | 1.25 |
| 13 | 23-Nordeoxycholic acid | NDCA | 26.11 | y = 2.641e+05x + 3981 | 1.00 | 0.25 - 200.00 | 0.25 |
| 14 | Dehydrocholic acid | DHCA | 15.82 | y = 1104x - 729.5 | 0.99 | 1.00 - 800.00 | 1.00 |
| 15 | 7,12-Diketolithocholic acid | 7,12-diketoLCA | 15.22 | y = 1.212e+04x + 1564 | 1.00 | 1.00 - 100.00 | 1.00 |
| 16 | 6,7-Diketolithocholic acid | 6,7-diketoLCA | 25.74 | y = 7.567e+04x + 4114 | 0.99 | 1.00 - 800.00 | 1.00 |
| 17 | α-Muricholic acid | alpha-MCA | 17.87 | y = 1.483e+05x + 1.159e+04 | 1.00 | 1.25 - 1000.00 | 1.25 |
| 18 | Ursocholic acid | UCA | 14.71 | y = 1.917e+05x + 1.019e+04 | 0.99 | 0.25 - 200.00 | 0.25 |
| 19 | β-Muricholic acid | beta-MCA | 18.81 | y = 1.431e+05x + 1.23e+04 | 0.99 | 1.25 - 1000.00 | 1.25 |
| 20 | Cholic acid | CA | 22.94 | y = 2.567e+05x - 2581 | 0.99 | 0.50 - 400.00 | 0.50 |
| 21 | Allocholic acid | ACA | 22.55 | y = 5.055e+05x + 1.147e+04 | 0.99 | 0.25 - 200.00 | 0.25 |
| 22 | 3β-Cholic acid | beta-CA | 17.24 | y = 5.379e+04x + 5.885e+06 | 0.99 | 125.00 - 2000.00 | 125.00 |
| 23 | Glycolithocholic acid | GLCA | 29.16 | y = 1.027e+04x - 878.6 | 0.99 | 1.00 - 800.00 | 1.00 |
| 24 | Glycohyodeoxycholic acid | GHDCA | 17.43 | y = 4331x - 135.7 | 0.99 | 1.00 - 800.00 | 1.00 |
| 25 | Glycochenodeoxycholic acid | GCDCA | 23.61 | y = 1.09e+04x + 17.58 | 1.00 | 1.25 - 1000.00 | 1.25 |
| 26 | Glycoursodeoxycholic acid | GUDCA | 17.11 | y = 8780x - 15.91 | 0.99 | 1.00 - 800.00 | 1.00 |
| 27 | Glycodeoxycholic acid | GDCA | 24.52 | y = 8724x - 1776 | 0.99 | 1.00 - 800.00 | 1.00 |
| 28 | Lithocholic acid-3-sulfate | LCA-3S | 28.51 | y = 1.078e+04x - 3729 | 0.99 | 1.00 - 800.00 | 1.00 |
| 29 | Glycocholic acid | GCA | 17.39 | y = 7400x + 260 | 0.99 | 1.25 - 1000.00 | 1.25 |
| 30 | Taurolithocholic acid | TLCA | 27.13 | y = 7212x - 2546 | 1.00 | 1.00 - 800.00 | 1.00 |
| 31 | Taurohyodeoxycholic acid + Taurohyocholic acid | THDCA+TUDCA | 14.58 | y = 1974x + 46.54 | 0.99 | 2.50 - 2000.00 | 2.50 |
| 32 | Taurodeoxycholic acid | TDCA | 22.04 | y = 4838x - 1123 | 1.00 | 1.25 - 1000.00 | 1.25 |
| 33 | Taurochenodeoxycholic acid | TCDCA | 20.66 | y = 6770x - 1087 | 1.00 | 1.25 - 1000.00 | 1.25 |
| 34 | Taurocholic acid | TCA | 15.19 | y = 5487x + 333.7 | 1.00 | 1.25 - 1000.00 | 1.25 |
| 35 | Tauro-α-muricholic acid | T-alpha-MCA | 9.46 | y = 2380x - 66.45 | 1.00 | 1.25 - 1000.00 | 1.25 |
| 36 | Taurohyocholic acid | THCA | 12.50 | y = 5991x - 1332 | 1.00 | 0.50 - 400.00 | 0.50 |
| 37 | Tauro-β-muricholic acid | T-beta-MCA | 9.82 | y = 4392x - 1099 | 1.00 | 1.25 - 1000.00 | 1.25 |
| 38 | Chenodeoxycholic acid-glucuronide | CDCA-G | 22.62 | y = 1407x - 942.6 | 0.99 | 4.00 - 3200.00 | 4.00 |
| 39 | Glycochenodeoxycholic acid-3-sulfate | GCDCA-3S | 18.07 | y = 9.398e+04x - 3.422e+04 | 1.00 | 1.25 - 1000.00 | 1.25 |
| 40 | Chenodeoxycholic acid-3-sulfate | CDCA-3S | 23.73 | y = 3.442e+05x - 5.569e+04 | 0.99 | 0.63 - 500.00 | 0.63 |
| 41 | Taurodehydrocholic acid | TDHCA | 6.97 | y = 2.101e+05x - 2.604e+04 | 1.00 | 0.63 - 500.00 | 0.63 |
| 42 | Isochenodeoxycholic acid | isoCDCA | 25.16 | y = 1.279e+05x + 4363 | 1.00 | 0.63 - 500.00 | 0.63 |
| 43 | Glycolithocholic acid-3-sulfate | GLCA-3S | 23.76 | y = 428.2x - 1121 | 0.99 | 2.50 - 2000.00 | 2.50 |
| 44 | Hyocholic acid | HCA | 21.15 | y = 2.883e+05x - 5018 | 1.00 | 0.63 - 125.00 | 0.63 |
| 45 | 7α-Hydroxy-3-oxo-4-cholenoic acid | 7OH3OCholA | 30.48 | y = 9.219e+04x - 2.749e+04 | 0.99 | 1.25 - 1000.00 | 1.25 |
| 46 | Taurolithocholic acid-3-sulfate | TLCA-3S | 20.91 | y = 3.474e+04x - 9.912e+04 | 1.00 | 6.25 - 5000.00 | 6.25 |
| 47 | Taurocholic acid-3-sulfate | TCA-3S | 11.88 | y = 2.32e+04x - 5277 | 1.00 | 2.50 - 2000.00 | 2.50 |
| 48 | Glycine-β-muricholic acid conjugate | Gly-MCA | 12.84 | y = 1.322e+04x + 2466 | 0.99 | 2.50 - 2000.00 | 2.50 |
| 49 | Glycocholic acid-3-sulfate | GCA-3S | 13.68 | y = 7.732e+04x - 6136 | 1.00 | 1.25 - 1000.00 | 1.25 |
| 50 | Glycohyocholic acid | GHCA | 15.13 | y = 2.159e+04x - 328.5 | 0.99 | 1.25 - 500.00 | 1.25 |
| 51 | Tauro-ω-muricholic acid | T-omega-MCA | 8.81 | y = 1.674e+04x - 4407 | 1.00 | 1.25 - 1000.00 | 1.25 |
| 52 | Taurochenodeoxycholic acid-3-sulfate | TCDCA-3S | 15.70 | y = 5808x - 9551 | 1.00 | 6.25 - 5000.00 | 6.25 |
| 53 | 3,7-Dihydroxycholan-24-oic acid | DiOH24CA | 28.11 | y = 2014x - 1036 | 1.00 | 2.50 - 500.00 | 2.50 |

**Table S2** The mobile phase gradient elution condition

| Time (min) | A (%) | B (%) |
| --- | --- | --- |
| 0 | 80 | 20 |
| 13 | 60 | 40 |
| 20 | 40 | 60 |
| 24 | 2 | 98 |
| 30 | 2 | 98 |
| 31 | 80 | 20 |
| 35 | 80 | 20 |

**Table S3** The bile acid standards and internal standards of bile acid targeted metabolomics

| Compound name | Manufacturer | Product number |
| --- | --- | --- |
| Isolithocholic acid (ILCA) | Germany, Cayman | 29545 |
| Chenodeoxycholic acid (CDCA) | Germany, Cayman | 10011286 |
| Deoxycholic acid (DCA) | Germany, Cayman | 20756 |
| Isodeoxycholic acid (IDCA) | Germany, Cayman | 29890 |
| *ω*-Muricholic acid (*ω*-MCA) | Germany, Cayman | 20292 |
| *α*-Muricholic acid (*α*-MCA) | Germany, Cayman | 20291 |
| *β*-Muricholic acid (*β*-MCA) | Germany, Cayman | 20287 |
| Glycoursodeoxycholic acid (GUDCA) | Germany, Cayman | 21698 |
| Glycine-*β*-muricholic Acid (G*β*MA) | Germany, Cayman | 9003230 |
| Lithocholic Acid 3-sulfate (3-SLCA) | Germany, Cayman | 20676 |
| Glycohyocholic acid (GHCA) | Germany, Cayman | 22670 |
| Taurolithocholic acid (TLCA) | Germany, Cayman | 17275 |
| Tauro-*ω*-muricholic acid (T-*ω*-MCA) | Germany, Cayman | 28842 |
| Tauro-*α*-muricholic acid (T-*α*-MCA) | Germany, Cayman | 20288 |
| Taurohyocholic acid (THCA) | Germany, Cayman | 22669 |
| Taurocholic acid (TCA) | Germany, Cayman | 16215 |
| Cholic Acid 7-sulfate (7-SCA) | Germany, Cayman | 9002532 |
| 23-Nordeoxycholic acid (NDCA) | Germany, Cayman | 30837 |
| Hyodeoxycholic acid (HDCA) | America, GLPBIO | GN10489 |
| Lithocholic acid (LCA) | China, Shanghai Yuanye Biotechnology Co., LTD | B28100 |
| Ursodeoxycholic acid (UDCA) | China, Shanghai Yuanye Biotechnology Co., LTD | B21405 |
| Cholic acid (CA) | China, Shanghai Yuanye Biotechnology Co., LTD | B20274 |
| Glycochenodeoxycholic Acid (GCDCA) | China, Shanghai Yuanye Biotechnology Co., LTD | B24724 |
| Glycodeoxycholic acid (GDCA) | China, Shanghai Yuanye Biotechnology Co., LTD | B32956 |
| Glycocholic acid (GCA) | China, Shanghai Yuanye Biotechnology Co., LTD | B27078 |
| Taurochenodeoxycholic acid (TCDCA) | China, Shanghai Yuanye Biotechnology Co., LTD | B20919 |
| Tauroursodeoxycholic acid (TUDCA) | China, Shanghai Yuanye Biotechnology Co., LTD | B20921 |
| Cholic acid-*d*_4_ | Germany, Cayman | 20849 |
| Chenodeoxycholic acid-*d*_4_ | Germany, Cayman | 20848 |
| Deoxycholic acid-*d*_4_ | Germany, Cayman | 20851 |

**Table S4** The mass spectrum parameters of bile acids

| Compound name | Q1  (Da) | Q3  (Da) | DP  (V) | CE  (eV) | RT  (min) | Internal  Standard |
| --- | --- | --- | --- | --- | --- | --- |
| Lithocholic acid (LCA) | 375.2 | 375.2 | -196 | -11 | 25.02 | LCA-*d*_4_ |
| Isolithocholic acid (ILCA) | 375.2 | 375.2 | -196 | -11 | 24.10 | LCA-*d*_4_ |
| 23-Nordeoxycholic acid (NDCA) | 377.1 | 377.1 | -179 | -14 | 19.55 | CDCA-*d*_4_ |
| Chenodeoxycholic acid (CDCA) | 391.2 | 391.2 | -178 | -10 | 21.19 | CDCA-*d*_4_ |
| Deoxycholic acid (DCA) | 391.2 | 391.2 | -168 | -16 | 21.70 | DCA-*d*_4_ |
| Isodeoxycholic acid (IDCA) | 391.2 | 391.2 | -178 | -10 | 24.20 | DCA-*d*_4_ |
| Ursodeoxycholic acid (UDCA) | 391.2 | 391.2 | -178 | -10 | 17.81 | UDCA-*d*_4_ |
| Hyodeoxycholic acid (HDCA) | 391.2 | 391.2 | -178 | -10 | 17.92 | CDCA-*d*_4_ |
| Cholic acid (CA) | 407.2 | 407.2 | -196 | -16 | 17.23 | CA-*d*_4_ |
| *ω*-Muricholic acid (*ω*-MCA) | 407.2 | 407.2 | -196 | -16 | 13.73 | CA-*d*_4_ |
| *α*-Muricholic acid (*α*-MCA) | 407.2 | 407.2 | -196 | -16 | 14.26 | CA-*d*_4_ |
| *β*-Muricholic acid (*β*-MCA) | 407.2 | 407.2 | -196 | -16 | 14.95 | CA-*d*_4_ |
| Glycochenodeoxycholic acid (GCDCA) | 448.3 | 448.3 | -100 | -20 | 17.79 | GCDCA-*d*_4_ |
| Glycoursodeoxycholic acid (GUDCA) | 448.3 | 448.3 | -100 | -20 | 13.92 | GCDCA-*d*_4_ |
| Glycodeoxycholic acid (GDCA) | 448.3 | 448.3 | -140 | -10 | 18.40 | GCDCA-*d*_4_ |
| Lithocholic acid 3-sulfate (3-SLCA) | 455.2 | 455.2 | -175 | -10 | 21.50 | LCA-*d*_4_ |
| Glycine-*β*-muricholic acid (G*β*MA) | 464.2 | 464.2 | -180 | -11 | 11.01 | GCA-*d*_4_ |
| Glycohyocholic acid (GHCA) | 464.2 | 464.2 | -180 | -11 | 12.43 | GCA-*d*_4_ |
| Glycocholic acid (GCA) | 464.2 | 464.2 | -180 | -11 | 13.90 | GCA-*d*_4_ |
| Taurolithocholic acid (TLCA) | 482.3 | 482.3 | -100 | -20 | 19.41 | LCA-*d*_4_ |
| Cholic acid 7-sulfate (7-SCA) | 487.2 | 487.2 | -208 | -10 | 13.22 | TCA-*d*_4_ |
| Taurochenodeoxycholic acid (TCDCA) | 498.2 | 124.0 | -166 | -12 | 15.04 | GCDCA-*d*_4_ |
| Tauroursodeoxycholic acid (TUDCA) | 498.2 | 124.0 | -164 | -10 | 11.30 | GCDCA-*d*_4_ |
| Tauro-*ω*-muricholic acid (T-*ω*-MCA) | 514.1 | 124.0 | -145 | -23 | 7.91 | TCA-*d*_4_ |
| Tauro-*α*-muricholic acid  (T-*α*-MCA) | 514.2 | 124.0 | -145 | -23 | 8.28 | TCA-*d*_4_ |
| Taurohyocholic acid (THCA) | 514.2 | 514.2 | -150 | -16 | 9.96 | TCA-*d*_4_ |
| Taurocholic acid (TCA) | 514.2 | 124.0 | -111 | -11 | 11.62 | TCA-*d*_4_ |

**Table S5** The primer sequences of qRT-PCR

| Genes | Forward (5′-3′) | Reverse (5′-3′) |
| --- | --- | --- |
| mouse-GAPDH | TTCCAGTATGACTCTACCCACGGCA | GCACCAGCATCACCCCATTTG |
| mouse-FXR | ACAGCGAAGGGCGTGACTTG | GGTCTGTTGGTCTGCCGTGAG |
| *L. reuteri* | TTAAGGATGCAAACCCGAAC | CCTTGTCACCTGGAACCACT |

**Table S6** Baseline of healthy controls (HCs) and ALD patients

| Variables | HCs (*n* = 22) | ALD (*n* = 22) | *P* value |
| --- | --- | --- | --- |
| Age | 57.2±7.7 | 57.7±8.5 | 0.8240 |
| Gender (male) | 20, 90.9% | 20, 90.9% | 1.0000 |
| BMI (kg/m^2^) | 25.3±3.7 | 22.4±4.2 | 0.0200 |
| Smoking | 9, 40.9% | 13, 59.1% | 0.2380 |
| TP (g/L) | 75.4±7.4 | 72.0±6.5 | 0.1580 |
| TBA (μM) | 2.4±1.6 | 20.2±18.3 | 0.0010 |
| CHE (U/L) | 8266.1±2029.6 | 6019.8±3212.3 | 0.0080 |
| ALB (g/L) | 44.8±3.8 | 39.2±9.2 | 0.0100 |
| GLB (g/L) | 30.2±5.6 | 35.2±7.7 | 0.0350 |
| AG (ratio) | 1.5±0.3 | 1.1±0.4 | 0.0010 |
| TBIL (μM) | 9.7±4.6 | 25.2±16.4 | < 0.0001 |
| DBIL (μM) | 6.5±3.8 | 16.7±14.2 | 0.0060 |
| IBIL (μM) | 3.9±2.6 | 10.6±5.9 | < 0.0001 |
| ALT (U/L) | 28.7±17.3 | 41.9±30.3 | 0.0800 |
| AST (U/L) | 20.8±9.4 | 68.2±41.0 | < 0.0001 |
| GGT (U/L) | 19.9±9.5 | 179.0±144.0 | < 0.0001 |
| TC (mM) | 4.2±1.1 | 4.7±1.3 | 0.1600 |
| TG (mM) | 2.1±1.1 | 2.1±1.0 | 0.9580 |
| LDLC (mM) | 1.8±0.9 | 2.5±1.0 | 0.0200 |
| HDLC (mM) | 1.7±0.5 | 1.4±0.6 | 0.0700 |
| GLU (mM) | 4.8±0.5 | 6.4±2.2 | 0.0200 |
| Alcohol consumption (mL/week) | 0 | 582.6±363.9 | < 0.0001 |
| Drinking duration (years) | 0 | 35.2±10.8 | < 0.0001 |
| Stage II (Alcoholic fatty liver)**, n (%)** | 0 | 12 **(54.5)** | < 0.0001 |
| Stage III (Alcoholic hepatitisr)**, n (%)** | 0 | 10 **(45.5)** | < 0.0001 |

Note: TP: total protein; TBA: total bile acids; CHE: cholinesterase; ALB: albumin; GLB: globulin; AG: albumin/globulin ratio; TBIL: total bilirubin; DBIL: direct bilirubin; IBIL: indirect bilirubin; ALT: alanine aminotransferase; AST: aspartate aminotransferase; GGT: gamma-glutamyl transferase; TC: total cholesterol; TG: triglycerides; LDLC: low-density lipoprotein cholesterol; HDLC: high-density lipoprotein cholesterol; GLU: Glucose.

**Table S7** *L. reuteri*-enriched metabolites identified by targeted metabolomics (T700).

| Name | Class | log_2_(FC) | *P* value |
| --- | --- | --- | --- |
| Phe-Ala | Small peptide | 19.038 | 6.19E-07 |
| Aceglutamide | Amino acid derivatives | 17.662 | 3.01E-02 |
| Thiamine pyrophosphate | CoEnzyme and vitamins | 17.500 | 2.82E-04 |
| D-Erythrose 4-phosphate | Phosphate sugars | 17.236 | 2.34E-05 |
| Dihydrothymine | Nucleotide and its metabolomics | 17.111 | 8.81E-04 |
| Apigenic acid | FFA | 15.483 | 3.77E-04 |
| N8-Acetylspermidine | Amines | 15.295 | 5.06E-07 |
| L-NIO-hydrochloride | Amino acid derivatives | 13.828 | 1.46E-04 |
| 1-(9Z,12Z-octadecadienoyl)-sn-glycero-3-phosphocholine | LPC | 12.767 | 2.90E-03 |
| L-Leucic acid | Organic acid and its derivatives | 5.297 | 2.87E-05 |
| 3-phenyllactic acid | Organic acid and its derivatives | 4.863 | 2.89E-04 |
| Indolelactic acid | Indole and its derivatives | 4.180 | 3.97E-08 |
| Glutamine | Amino acids | 3.832 | 5.61E-07 |
| Sorbitol | Sugar alcoholss | 3.799 | 8.72E-04 |
| Mannitol | Sugar alcoholss | 3.770 | 5.01E-04 |
| N-Acetylthreonine | Amino acid derivatives | 3.593 | 5.92E-06 |
| N-Acetylcadaverine | Amines | 3.415 | 8.30E-07 |
| Hydroxyphenyllactic acid | Organic acid and its derivatives | 3.365 | 3.31E-05 |
| L-citrulline | Amino acids | 2.857 | 6.67E-03 |
| Galactose 1-phosphate | Phosphate sugars | 2.776 | 1.39E-05 |
| Ornithine | Amino acids | 2.719 | 1.85E-06 |
| L-Lactic acid | Organic acid and its derivatives | 2.468 | 4.23E-05 |
| D-Glucose 6-phosphate | Phosphate sugars | 1.988 | 3.07E-05 |
| Deoxycholic acid | Bile acids | 1.974 | 3.38E-03 |
| Guanine | Nucleotide and its metabolomics | 1.733 | 1.37E-04 |
| N-Propionylglycine | Amino acid derivatives | 1.689 | 8.30E-04 |
| Isoursodeoxycholic acid | Bile acids | 1.652 | 6.64E-03 |
| Adenine | Nucleotide and its metabolomics | 1.558 | 4.48E-07 |
| 2-Picolinic Acid | Pyridines and derivatives | 1.508 | 5.13E-05 |
| N-Acetyl-L-glutamic acid | Amino acid derivatives | 1.474 | 2.60E-05 |
| Vitamin B3 | CoEnzyme and vitamins | 1.435 | 1.07E-04 |
| N-Acetylaspartate | Amino acid derivatives | 1.415 | 1.03E-04 |
| Oxypurinol | Nucleotide and its metabolomics | 1.375 | 1.62E-03 |
| N-Acetylputrescine | Organic acid and its derivatives | 1.254 | 7.00E-05 |
| N-Acetylasparagine | Amino acid derivatives | 1.238 | 1.01E-04 |
| Xyluose-5-phosphate | Phosphate sugars | 1.234 | 7.38E-03 |
| Allocholic acid | Bile acids | 1.233 | 9.11E-03 |
| Cholic acid | Bile acids | 1.034 | 7.09E-04 |
| O-Acetyl-L-homoserine | Amino acid derivatives | 0.894 | 1.92E-05 |
| N-Acetyl-beta-alanine | Amino acid derivatives | 0.783 | 5.26E-04 |
| Uracil | Nucleotide and its metabolomics | 0.780 | 2.18E-03 |
| 2-hydroxyisovaleric Acid | Organicacid and its derivatives | 0.760 | 2.42E-04 |
| N-acetylalanine | Amino acid derivatives | 0.745 | 1.09E-02 |
| N-formylkynurenine | Organic acid and its derivatives | 0.681 | 2.23E-03 |
| Glycylphenylalanine | Small peptide | 0.568 | 1.31E-03 |
| Ferulic Acid | Organic acid and its derivatives | 0.541 | 2.00E-02 |
| 1-Methyladenine | Nucleotide and its metabolomics | 0.512 | 3.02E-02 |
| Benzenepropanoic acid | Organic acid and its derivatives | 0.497 | 1.30E-02 |
| Tryptophol | Indole and its derivatives | 0.476 | 4.62E-04 |
| Hypoxanthine | Nucleotide and its metabolomics | 0.463 | 9.07E-03 |
| 2-Hydroxyglutaric acid | Organic acid and its derivatives | 0.424 | 1.87E-04 |
| Octanoic Acid | FFA | 0.422 | 1.62E-02 |
| Acetic Acid | SCFA | 0.420 | 2.28E-03 |
| Gly-Trp | Small peptide | 0.396 | 3.77E-04 |
| Succinic Acid | Organic acid and its derivatives | 0.384 | 4.47E-03 |
| Uric acid | Imidazopyrimidines | 0.381 | 3.49E-02 |
| N-acetyltryptophan | Amino acid derivatives | 0.362 | 4.22E-02 |
| Glycyl-DL-phenylalanine | Amino acid derivatives | 0.357 | 1.42E-02 |
| Histamine | Amines | 0.276 | 9.35E-05 |
| Gly-Gly | Small peptide | 0.250 | 4.22E-04 |
| beta-indole-3-acetic acid | Indole and its derivatives | 0.208 | 2.77E-02 |
| 2-Aminoisobutyric Acid | Amino acid derivatives | 0.208 | 1.47E-02 |
| Propionic Acid | SCFA | 0.194 | 3.91E-02 |
| Indole-3-Carboxaldehyde | Indole and its derivatives | 0.193 | 2.82E-02 |
| Glycerol 3-phosphate | Phosphoric acids | 0.173 | 4.06E-02 |
| Gly-Glu | Small peptide | 0.170 | 6.37E-02 |
| 4-Acetamidobutyric Acid | Amino acid derivatives | 0.169 | 7.68E-04 |
| Glycyl-dl-serine | Small peptide | 0.133 | 3.77E-02 |
| Creatinine | Organic acid and its derivatives | 0.125 | 1.17E-03 |
| L-Alanine | Amino acids | 0.113 | 3.13E-02 |
| vitamin B1 | CoEnzyme and vitamins | 0.113 | 2.73E-03 |
| Histidine | Amino acids | 0.109 | 4.67E-02 |
| Tyramine | Benzene and substituted derivatives | 0.107 | 3.67E-02 |
| Threonine | Amino acids | 0.107 | 4.17E-02 |
| L-Proline | Amino acids | 0.096 | 4.79E-03 |
| L-Glutamic acid | Amino acids | 0.034 | 4.45E-02 |

Note: *L. reuteri* supernatant compared with heat‑killed *L. reuteri* (HKL) supernatant, log_2_(FC) and *P* value are shown.

**Table S8** Screening of potential FXR agonists from *L. reuteri*-enriched metabolites by molecular docking

| Compound Name | Binding Site (Grid) | Glide gscore (kcal/mol) |
| --- | --- | --- |
| Thiamine-pyrophosphate | grid_002 | -8.25740 |
| Indolelactic-acid | grid_001 | -8.02102 |
| Phe-Ala | grid_002 | -7.25416 |
| Dihydrothymine | grid_001 | -7.06539 |

**Table S9** Statistical analysis of serum bile acid levels of HCs and ALD

| Compound name | Fold change (ALD/HCs) | *P* value |
| --- | --- | --- |
| 7OH3OCholA | 1.98 | 4.94E-04 |
| alpha-MCA | 2.04 | 4.29E-03 |
| GCDCA | 8.14 | 6.02E-03 |
| DCA | 2.98 | 6.82E-03 |
| CDCA-G | 6.75 | 9.03E-03 |
| GCA | 11.56 | 9.18E-03 |
| TCDCA | 45.42 | 9.55E-03 |
| GHCA | 7.67 | 1.23E-02 |
| CA | 6.55 | 1.38E-02 |
| LCA-3S | 3.24 | 1.39E-02 |
| GDCA | 3.23 | 1.92E-02 |
| TCA | 60.46 | 1.94E-02 |
| CDCA | 4.18 | 2.17E-02 |
| GCDCA-3S | 11.21 | 2.64E-02 |
| LCA | 0.66 | 3.77E-02 |
| THCA | 37.53 | 3.97E-02 |
| DiOH24CA | 4.48 | 4.04E-02 |
| GCA-3S | 11.21 | 4.24E-02 |
| CDCA-3S | 55.86 | 5.92E-02 |
| T-omega-MCA | 8.07 | 6.48E-02 |
| TDCA | 5.11 | 7.36E-02 |
| alloLCA | 0.24 | 8.46E-02 |
| TLCA | 2.55 | 8.48E-02 |
| 7-ketoLCA | 4.61 | 9.04E-02 |
| GUDCA | 2.39 | 9.34E-02 |
| THDCA+TUDCA | 37.19 | 1.04E-01 |
| HCA | 17.92 | 1.06E-01 |
| TCDCA-3S | 48.56 | 1.26E-01 |
| NDCA | 11.74 | 1.27E-01 |
| GLCA | 1.68 | 1.38E-01 |
| GHDCA | 15.29 | 1.41E-01 |
| beta-UDCA | 0.24 | 1.47E-01 |
| 12-ketoLCA | 1.71 | 1.69E-01 |
| 7,12-diketoLCA | 73.14 | 1.83E-01 |
| TCA-3S | 2235.80 | 1.85E-01 |
| UDCA | 0.58 | 2.80E-01 |
| TLCA-3S | 1.39 | 4.05E-01 |
| isoCDCA | 0.70 | 4.24E-01 |
| HDCA | 1.88 | 4.52E-01 |
| ACA | 1.27 | 5.50E-01 |
| UCA | 1.41 | 6.52E-01 |
| NCA | 0.79 | 6.52E-01 |
| isoLCA | 0.89 | 7.62E-01 |
| beta-MCA | 1.18 | 8.07E-01 |
| 6-ketoLCA | 1.16 | 8.86E-01 |
| GLCA-3S | 1.03 | 9.37E-01 |

**Table S10** Adjusted *P* value of key differential metabolites and gut microbiota between HCs and ALD group using ANCOVA

| Variable | Adjusted *P* value |
| --- | --- |
| CA | 0.441 |
| DCA | 0.461 |
| NDCA | 0.756 |
| *Lactobacillus* | 0.813 |
| *Lactobacillus reuteri* | 0.298 |

Note: An adjusted *P* value > 0.05 indicates no statistically significant after controlling for confounders.

**Table S11** Differential bile acid in liver of NOR and MOD mice

| Compound name | Fold change (MOD/NOR) | *P* value |
| --- | --- | --- |
| DCA | 3.12 | 1.66E-06 |
| NDCA | 8.62 | 1.05E-04 |
| T-omega-MCA | 1.75 | 3.52E-04 |
| beta-MCA | 3.42 | 3.78E-04 |
| CA | 9.33 | 9.34E-04 |
| 7-SCA | 0.41 | 1.63E-03 |
| GUDCA | 3.29 | 2.23E-03 |
| GBCA | 6.05 | 2.95E-03 |
| GCA | 3.26 | 6.78E-03 |
| omega-MCA | 3.23 | 8.40E-03 |
| GCDCA | 1.63 | 3.80E-02 |
| TLCA | 0.62 | 4.81E-02 |

**Fig. S1** Alterations in gut microbiota and barrier function in ALD mice. (A) Alpha diversity of fecal samples between NOR and MOD mice (*n* = 8). (B) Principal coordinates analysis (PcoA) plot of fecal samples between NOR and MOD mice. (C) Circos plot of fecal samples between NOR and MOD mice. (D) H&E staining of small intestine tissues in NOR and MOD mice. (E) Plasma lipopolysaccharide (LPS) levels in NOR and MOD mice (*n* = 8). (F) Immunohistochemical staining of Occludin in small intestine tissues in NOR and MOD mice. NOR: normal group; MOD: model group. Compared with the NOR, ^##^*P <* 0.01, ^###^*P <* 0.001.

**Fig. S2** *L. reuteri* administration effectively restored the impaired intestinal barrier in ALD mice. (A) Immunofluorescence staining of Occludin in intestinal tissues. (scale bar = 500 µm, *n* = 3). (B) Immunofluorescence staining of ZO-1 in intestinal tissues. (scale bar = 500 µm, *n* = 3). (C) Immunofluorescence staining of Claudin-1 in intestinal tissues. (scale bar = 500 µm, *n* = 3). NOR: normal group; MOD: model group; Lr: ALD mice treated with *L. reuteri*. Compared with NOR, ^#^*P <* 0.05, ^##^*P <* 0.01; compared with MOD, **^*^***P <* 0.05, **^**^***P <* 0.01.

**Fig. S3** Metabolomic profiling of fecal contents following *L. reuteri* administration using the T700 platform. (A) Partial least squares discriminant analysis (PLS-DA) score plot showing the separation among different groups. (B) Permutation test (200 times) for model validation. (C) Hierarchical tree map illustrating the structural classes of differential metabolites between the NOR and MOD groups. (D) Hierarchical tree map illustrating the structural classes of differential metabolites between the MOD and Lr groups. NOR: normal group; MOD: model group; Lr: ALD mice treated with *L. reuteri*.

**Fig. S4** Immunofluorescence staining of GPCR43 and AHR in intestinal tissues. (scale bar = 500 µm，*n* = 3). NOR: normal group; MOD: model group; Lr: ALD mice treated with *L. reuteri*. Compared with MOD, ^*^*P <* 0.05.

**Fig. S5** Western blot replications of cell and mice liver. (A) Western blot replications of HepG2 cell (*n* = 4). (B) Western blot replications of L02 cell (*n* = 4). (C) Western blot replications in mice liver of *L. reuteri* (*n* = 4). (D) Western blot replications in mice liver of ECH (*n* = 4). Lr: ALD mice or cell treated with *L. reuteri*; HKL: ALD mice or cell treated with heat-killed *L. reuteri*. ECH: ALD mice treated with ECH (200 mg/kg).

**Fig. S6** Analysis of potential metabolites enriched by *Lactobacillus reuteri* (*L. reuteri*) using T700. (A) Principal component analysis (PCA) score plot between groups. (B) Heatmap of all detected compounds across samples. (C) Volcano plot of all detected compounds. (D) Hierarchical tree map of the structural classes of potential metabolites enriched by *L. reuteri.* (E) Sankey and heatmap diagram of potential metabolites enriched by *L. reuteri.* Lr: the supernatant of *L. reuteri*；HKL: the supernatant of heat-killed *L. reuteri.*

**Fig. S7** Association analysis of gut microbiota, metabolites, and disease indices in human samples. (A) Spearman correlation heatmap showing pairwise relationships among differential metabolites, gut bacterial genera, and baseline clinical parameters. Red indicates positive correlation; blue indicates negative correlation. Color intensity represents correlation strength. ^*^*P <* 0.05, and ^**^*P <* 0.01. (B) Correlation network diagram constructed using only variables with significant correlations. Nodes represent individual variables; edges represent significant correlations; edge thickness reflects correlation strength; yellow edges indicate positive correlations; grey edges indicate negative correlations. Note: ALD: alcoholic liver disease; TP: total protein; TBA: total bile acids; CHE: cholinesterase; ALB: albumin; GLB: globulin; AG: albumin/globulin ratio; TBIL: total bilirubin; DBIL: direct bilirubin; IBIL: indirect bilirubin; ALT: alanine aminotransferase; AST: aspartate aminotransferase; GGT: gamma-glutamyl transferase; TC: total cholesterol; TG: triglycerides; LDLC: low-density lipoprotein cholesterol; HDLC: high-density lipoprotein cholesterol; GLU: Glucose.

**Fig. S8** Impact of bile acid administration on physiological parameters in ALD mice. (A) Body weight and liver index in each group (*n* = 8). (B) ALT, AST, and TG levels in each group (*n* = 8). (C) GSH-Px, SOD, and MDA levels in each group (*n* = 8). (D) IL-1β and TNF-α levels in each group (*n* = 8). (E) H&E staining of liver and small intestine tissues, and Oil red O staining in each group (*n* = 3), scale bar = 100 µm. NOR: normal group; MOD: model group; CA: ALD mice treated with CA; DCA: ALD mice treated with DCA. Compared with the NOR, ^#^*P <* 0.05, ^##^*P <* 0.01, ^###^*P <* 0.001, ^####^*P <* 0.0001; compared with the MOD, ^*^*P <* 0.05, ^***^*P <* 0.001.

**Fig. S9** Impact of bile acid administration on physiological parameters in *Fxr*^-/-^normal mice. (A) Body weight, liver index, ALT, AST, GSH-Px, MDA, TNF-α, IL-1β levels in each group (*n* = 10). (B) H&E staining of small intestine tissues and Oil red O staining of liver (*n* = 3), scale bar =100 µm. NOR: normal group; NOR+CA: normal mice treated with CA; NOR+DCA: normal mice treated with DCA. Compared with the NOR, ^#^*P <* 0.05, ^##^*P <* 0.01, ^###^*P <* 0.001.

**Fig. S10** Echinacoside (ECH) promotes *L. reuteri* growth. (A) Growth curves of *L. reuteri* in standard GAM medium. (B) Growth curves of *L. reuteri* in modified GAM broth without glucose and soluble starch. Values with different lowercase letters indicate statistically significant differences among groups at the level of *P* < 0.05. Samples sharing at least one common lowercase letter have no significant difference, whereas those with completely different lowercase letters are significantly different.

**Fig. S11.** Regulatory effects of ECH on bile acid metabolism in ALD mice. Bar chart of liver bile acids in each group (*n* = 8). NOR: normal group; MOD: model group; ECH 50: ALD mice treated with ECH (50 mg/kg); ECH 100: ALD mice treated with ECH (100 mg/kg); ECH 200: ALD mice treated with ECH (200 mg/kg). Compared with the NOR, ^#^*P <* 0.05, ^##^*P <* 0.01, ^###^*P <* 0.001; compared with the MOD, **^*^***P <* 0.05, **^**^***P <* 0.01, and **^***^***P <* 0.001.

**Fig. S12.** Regulatory effects of ECH on gut microbiota in ALD mice. (A) Principal coordinates analysis (PCoA) plot of fecal samples. (C) Bubble plot of the genus level. NOR: normal group; MOD: model group; ECH 200: ALD mice treated with ECH (200 mg/kg).
